# Prenatal Air Pollution Exposure and Autism Spectrum Disorder in the ECHO Consortium

**DOI:** 10.1021/EHP.6c00106

**Published:** 2026-04-22

**Authors:** Akhgar Ghassabian, Aisha S. Dickerson, Yuyan Wang, Joseph M. Braun, Deborah H. Bennett, Lisa A. Croen, Kaja Z. LeWinn, Heather H. Burris, Rima Habre, Kristen Lyall, Jean A. Frazier, Hannah C. Glass, Stephen R. Hooper, Robert M. Joseph, Catherine J. Karr, Rebecca J. Schmidt, Chloe Friedman, Margaret R. Karagas, Annemarie Stroustrup, Jennifer K. Straughen, Anne L. Dunlop, Jody M. Ganiban, Leslie D. Leve, Rosalind J. Wright, Cindy T. McEvoy, Alison E. Hipwell, Angelo P. Giardino, Hudson P. Santos, Hannah Krause, Emily Oken, Carlos A. Camargo, Jiwon Oh, Christine Loftus, T. Michael O’Shea, Thomas G. O’Connor, Adam Szpiro, Heather E. Volk

**Affiliations:** † Department of Pediatrics, 12296 NYU Grossman School of Medicine, New York, New York 10016, United States; ‡ Department of Population Health, 12296 NYU Grossman School of Medicine, New York, New York 10016, United States; § Department of Epidemiology, Bloomberg School of Public Health, Johns Hopkins University, Baltimore, Maryland 21287, United States; ∥ Department of Epidemiology, 6752 Brown University School of Public Health, Providence, Rhode Island 02912, United States; ⊥ Department of Public Health Sciences, 12218 UC Davis School of Medicine, Sacramento, California 95817, United States; # Division of Research, 214681 Kaiser Permanente Northern California, Oakland, California 94612, United States; ○ Department of Psychiatry and Behavioral Sciences, University of California San Francisco, San Francisco, California 94143, United States; □ Department of Pediatrics, University of Pennsylvania Perelman School of Medicine, Philadelphia, Pennsylvania 19104, United States; △ Division of Neonatology, Children’s Hospital of Philadelphia, Philadelphia, Pennsylvania 19104, United States; ▽ Department of Population and Public Health Sciences, USC Keck School of Medicine, Los Angeles, California 90033, United States; ● A.J. Drexel Autism Institute, 6527 Drexel University, Philadelphia, Pennsylvania 19104, United States; ■ Eunice Kennedy Shriver Center, 12262 University of Massachusetts Chan Medical School, Worcester, Massachusetts 01655, United States; ▲ Department of Psychiatry, 12262 University of Massachusetts Chan Medical School, Worcester, Massachusetts 01655, United States; ▼ Department of Pediatrics, 12262 University of Massachusetts Chan Medical School, Worcester, Massachusetts 01655, United States; a Department of Neurology, University of California San Francisco, San Francisco, California 94143, United States; b Weill Institute for Neuroscience, University of California San Francisco, San Francisco, California 94143, United States; c Department of Pediatrics, UCSF Benioff Children’s Hospital, University of California San Francisco, San Francisco, California 94143, United States; d Department of Health Sciences, 6797 University of North Carolina School of Medicine, Chapel Hill, North Carolina 27599, United States; e Department of Anatomy and Neurobiology, 12259 Boston University Chobanian and Avedisian School of Medicine, Boston, Massachusetts 02118, United States; f Department of Pediatrics, University of Washington School of Medicine, Seattle, Washington 98195, United States; g Department of Environmental and Occupational Health Sciences, University of Washington School of Public Health, Seattle, Washington 98195, United States; h The MIND Institute, 8789 University of California Davis, Sacramento, California 95616, United States; i Lifecourse Epidemiology of Adiposity and Diabetes (LEAD) Center, 129263 University of Colorado Anschutz Medical Campus, Aurora, Colorado 80045, United States; j Department of Epidemiology, Geisel School of Medicine at Dartmouth, Hanover, New Hampshire 03755, United States; k Division of Neonatology, Cohen Children’s Medical Center, 5799 Northwell Health, New Hyde Park, New York 10022, United States; l Department of Pediatrics, Zucker School of Medicine at Hofstra/Northwell, Uniondale, New York 11549, United States; m Department of Occupational Medicine, Epidemiology and Prevention, Zucker School of Medicine at Hofstra/Northwell, Uniondale, New York 11549, United States; n Department of Public Health Sciences, Henry Ford Health, Detroit, Michigan 48202, United States; o Department of Obstetrics, Gynecology and Reproductive Biology, College of Human Medicine, Michigan State University, East Lansing, Michigan 48824, United States; p Department of Gynecology and Obstetrics, 1371 Emory University School of Medicine, Atlanta, Georgia 30322, United States; q Department of Psychological and Brain Sciences, 8367 George Washington University, Washington, DC 20052, United States; r Prevention Science Institute, 3265 University of Oregon, Eugene, Oregon 97403, United States; s Department of Public Health, 5925 Icahn School of Medicine at Mount Sinai, New York, New York 10029, United States; t Department of Pediatrics, Papé Pediatric Research Institute, 6684 Oregon Health and Science University, Portland, Oregon 97239, United States; u Department of Psychiatry, 6614 University of Pittsburgh, Pittsburgh, Pennsylvania 15260, United States; v Department of Pediatrics, Spencer Fox Eccles School of Medicine, 7060 University of Utah, Salt Lake City, Utah 84112, United States; w 5452 University of Miami, School of Nursing and Health Studies, Miami, Florida 33146, United States; x 12328 Vanderbilt University Medical Center, Nashville, Tennessee 37232, United States; y Department of Population Medicine, 731328 Harvard Medical School and Harvard Pilgrim Health Care Institute, Cambridge, Massachusetts 02115, United States; z Department of Emergency Medicine, Massachusetts General Hospital, Harvard Medical School, Boston, Massachusetts 02115, United States; 1 Division of Neonatal-Perinatal Medicine, University of North Carolina at Chapel Hill, Chapel Hill, North Carolina 27599, United States; 2 Department of Psychiatry, 6927 University of Rochester Medical Center, Rochester, New York 14642, United States; 3 Department of Neuroscience, 6927 University of Rochester Medical Center, Rochester, New York 14642, United States; 4 Department of Obstetrics and Gynecology, 6927 University of Rochester Medical Center, Rochester, New York 14642, United States; 5 Department of Biostatistics, University of Washington School of Public Health, Seattle, Washington 98195, United States; 6 Department of Mental Health, Johns Hopkins Bloomberg School of Public Health, Baltimore, Maryland 21205, United States; 7 Department of Environmental Health and Engineering, Johns Hopkins Bloomberg School of Public Health, Baltimore, Maryland 21205, United States

## Abstract

**BACKGROUND**: The relationship between prenatal exposure to low-level air pollution and child autism spectrum disorder (ASD) is unclear. **OBJECTIVE**: To examine associations of prenatal air pollution exposure with autism. **METHODS**: We analyzed data from 8,035 mother-child pairs from 44 United States cohorts in the Environmental influences on Child Health Outcomes (ECHO) Cohort. Fine particulate matter (PM_2.5_), nitrogen dioxide (NO_2_), and 8-h-max ozone (O_3_) levels were estimated at residential addresses during pregnancy. Parents rated children’s autism-related traits using the Social Responsiveness Scale (SRS) (mean age 9.4 years, SD = 3.6) and reported physician-diagnosed ASD. We examined associations of the three air pollutants with SRS scores (10th, 50th, and 90th quantiles) using quantile regression and with ASD diagnosis using logistic regression. Models were run within census divisions, and coefficients were pooled in a meta-analysis. **RESULTS**: Average (SD) pregnancy exposures were 9.3 μg/m^3^ (2.7) for PM_2.5_, 21.8 ppb (8.8) for NO_2_, and 40.3 ppb (5.5) for O_3_, with variations across census divisions. The median SRS T-score was 46 (IQR = 41 to 52), and 444 children (5.5%) had an ASD diagnosis. Higher PM_2.5_ was associated with higher SRS scores at the 10th quantile (β = 0.74, 95% CI: 0.09, 1.40) but not at the median or highest quantile. The association between PM_2.5_ and ASD diagnosis was highly heterogeneous, with associations present in the South Central, Mountain, and Pacific census divisions. Heterogeneity was also high in the association between NO_2_ and SRS at the median and only in the mid-Atlantic, West North Central, and South Atlantic census divisions. Higher O_3_ was associated with higher SRS scores at the median (β per IQR increment = 0.83, 95% CI: 0.05, 1.61) and highest quantile (β = 2.19, 95% CI: 0.06, 4.32) in the meta-analysis. Higher O_3_ also was associated with ASD. **DISCUSSION**: Associations with ASD outcomes were present even at low levels of air pollutants.

## Introduction

Ambient air pollution is a major environmental threat that contributes to mortality and morbidity.^1^ Emerging data suggest detectable effects of prenatal and early life exposure to air pollution on the brain via various mechanisms, including neuroinflammation,^2^ endocrine disruption,^3,4^ and epigenetic changes.^5^ Given the potential for these exposures to disrupt neurodevelopment, there have been recent calls for action by advocacy groups and stakeholders for better regulation of air pollution to protect brain development.^6^

Autism spectrum disorder (ASD) is a neurodevelopmental disorder characterized by communication difficulties and repetitive behaviors, with symptoms often recognized in the first two years of life. The prevalence of ASD has been rising in the United States (U.S.) and worldwide, with the etiology remaining largely unknown.^7^ Although ASD is highly genetic, the role of environmental risk factors is also discussed.^8^

Several epidemiologic investigations have examined early life exposure to ambient air pollutants as a possible modifiable factor for ASD. These pollutants include nitrogen oxides (NO_*x*_) produced during fossil fuel combustion in power plants or motor vehicles; particulate matter (PM), a mixture of solid particles and liquid droplets that originate from roads, fields, and fires, etc.; and ground-level ozone (O_3_), a byproduct of chemical reactions between NO_*x*_ and volatile organic compounds. Characterization of ASD and ASD-like symptoms varied largely across studies, from parental rating of symptoms in large birth cohorts to the use of electronic health records to determine diagnosis in case-control studies. Some of these studies confirmed the diagnosis using additional measures. Meta-analyses of these studies has shown that risk of autism is higher in children who were prenatally exposed to PM with an aerodynamic diameter less than 2.5 μm (PM_2.5_),^9–12^ but associations were weak for NO_*x*_ ^10,11^ and null for O_3_.^10,11^ In one meta-analysis, exposure to PM_2.5_ was associated with a higher risk of ASD in males,^12^ but few studies have examined sex differences. Although confounding by socioeconomic status and place of residence remains a concern, careful evaluation of residual confounding supports the causality of the association between air pollution and ASD.^13^

In 2024, based on abundant evidence regarding the health impacts of air pollution, the U.S. Environmental Protection Agency (EPA) set new National Ambient Air Quality Standards (annual mean of 9.0 μg/m^3^ for PM_2.5_, 53 parts per billion (ppb) for nitrogen dioxide (NO_2_), and 70 ppb for ground-level O_3_).^14^ Several areas in the U.S. currently have exposure levels at or below these standards; however, the question remains whether exposure at or below current standard levels has harmful effects on the brain and neurodevelopment, as suggested by studies with higher exposure levels.^15^ To address this question, we estimated PM_2.5_, NO_2_, and O_3_ levels at the residential addresses of pregnant participants in a large U.S. nationwide sample and examined the extent to which prenatal exposure to low-level air pollution was associated with ASD diagnosis or autism-related symptoms in children. We used both ASD diagnosis and autism-like symptoms as reported by parents and used the latter in quantile regression models to examine the potential impact of air pollution across the distribution of autism-related traits in the general population. The quantile regression approach enabled us to estimate associations across the range of autism-like behaviors instead of only one central aspect of the distribution, even in the presence of skewed outcome data, thereby supporting a systematic analysis of the entire spectrum of the outcome’s distribution for better understanding of the underlying mechanisms.^16^ We hypothesized that prenatal exposure to these pollutants would be associated with higher odds of ASD and increased autism-related symptoms. We also examined the interaction between air pollution exposure and child sex in relation to ASD.

## Methods

### Participants

We analyzed data collected as part of the National Institutes of Health Environmental influences on Child Health Outcomes (ECHO) Cohort.^17,18^ In ECHO, 20,595 pregnant participants from 48 cohorts provided residential address information between 2000 and 2016, which was used to estimate exposure to air pollution. From this group, information on child autism outcome was available for 14,158 participants, of whom 920 (6.5%) had a diagnosis of ASD as reported by parents. A total of 8,324 mother-child pairs from 44 cohorts had data on both air pollution exposure and child diagnosis of ASD and autism-related traits (2,364 children were excluded because they were born before 2000 or after 2016; 3,326 children had missing address data within that period). For families with more than one child in the study, we randomly selected one child for inclusion in this sample to ensure that the assumption of independence holds in the models, as siblings share genetic and environmental risk factors (*n* = 433). As a result, 8,035 mother-child pairs were included in all analyses (Figure S1). In this sample, 1,245 child participants were potentially at high risk for ASD based on enrollment in preterm cohorts (three cohorts), enrollment of siblings of children with autism (five cohorts), or case-control studies of autism (three studies).^19–21^ High-risk cohorts were primarily concentrated in the Pacific (*n* = 656 children from high-risk cohorts), New England (*n* = 127 children from high-risk cohorts), South Atlantic (*n* = 145 children from high-risk cohorts), and North Central regions (*n* = 199 children from high-risk cohorts). Other cohorts were broadly sampled from the general population (*n* = 6,790).

Among mother–child pairs with outcome data (*n* = 14,158), pregnant participants who were excluded because of missing information on residential addresses (and therefore air pollution) were less likely to report a history of any psychiatric disorder (33.9% vs 39.4%) than included participants. Children excluded were more likely to be Hispanic (22.1% vs 15.4%) and have a diagnosis of ASD (7.8% vs 5.5%) than the children included, but the two groups were comparable in autism-related traits. Mothers in the two groups were also comparable in birth year (22% of both groups were born before 2008), age (mean age in both groups, 30 years), and educational level (40% of participants excluded had educational level up to some college vs 37% of those included), and employment status (31% employed in excluded group vs 27% among those included).

The ECHO Cohort protocol was approved by the ECHO single Institutional Review Board (IRB) and/or the cohorts’ local IRBs. Written informed consent or parental/guardian permission with child assent was obtained before data collection.

### Air Pollution Exposure Assessment

An ensemble model that integrated multiple machine learning algorithms, including neural network, random forest, and gradient boosting, with a variety of predictor variables, including chemical transport models, was used to estimate daily exposure to PM_2.5_ and NO_2_, and 8-h max O_3_ exposures.^22,23^ These models covered the entire contiguous U.S. with daily predictions on 1 km level grid cells from 2000 to 2016. We estimated participants’ daily residential exposures by using history of residential addresses during pregnancy obtained with self-reported surveys (reported as *from* and *to* dates for each address). We matched the coordinates of each address with the nearest 1 km grid cell. For each address per participant, we assigned a date to determine when a participant lived at a particular location. We calculated the average exposure in each trimester and the entire pregnancy using daily estimates at residential addresses. Although there were differences across sites and participants in terms of timing and number of surveys, address data were harmonized and geocoded centrally by the ECHO Cohort.

### Autism Assessment

We used parent-report of child ASD diagnosis (yes/no) and autism-related traits (continuous score) as outcomes mean age = 9.4 years, standard deviation [SD] = 3.6). In surveys, parents/caregivers were asked about any physician-diagnosed ASD for their children. The autism case-control studies and familial autism cohorts combined autism diagnosis (as reported by parents or identified through the health records) with clinical assessments and gold standard measures (i.e., Autism Diagnostic Observation Schedule and Autism Diagnostic Interview-Revised).^19,21,24,25^ Other cohorts relied on parental rating of diagnosis only. Parents/caregivers also reported their children’s autism-like traits using the Social Responsiveness Scale (SRS), a 65-item parent-report scale that has high internal validity, reliability, and reproducibility^26,27^ and well-established psychometric properties in both clinical and general population samples.^28,29^ The SRS provides a quantitative measurement of an ASD-related phenotype in which higher scores indicate higher traits. For this analysis, we used either preschool (validated for children between 2 years 6 months and 4 years 6 months) or school-age (validated for children between 4 and 18 years) versions of the SRS raw scores. Results from the school-age version were prioritized if a child had both measures.

### Statistical Analyses

Missing data for covariates (Table 1) were imputed by using Multiple Imputation by Chained Equations (MICE).^30^ We included 8,035 mother-child pairs in all analyses after multiple imputation of missing covariate data. We examined descriptive statistics for exposures, outcomes, and covariates among cohorts in the pooled sample and across nine census divisions in the U.S. We observed substantial geographic heterogeneity in exposure levels and outcomes (Table S1). Therefore, we first ran the models within census divisions and pooled coefficients in a meta-analysis to account for geographical variability^31^ using inverse variance weighting of the division-specific estimates based on either fixed effects or random effects models, depending on the heterogeneity of the estimates.^32^ Forest plots were generated to display each division’s contribution to each summary estimate. Potential heterogeneity between divisions was assessed by using the I^2^, which measures the percentage of variability in estimates that is due to heterogeneity rather than chance, and Cochran’s Q test *p* value, which checks for significant heterogeneity among the divisions. If the Cochran’s Q test *p* value was nonsignificant, suggesting low heterogeneity among the divisions, pooled coefficients were derived from fixed-effect models. If the Cochran’s Q test *p* value was significant (high heterogeneity), coefficients were derived from random-effect models. We combined East and West South-Central divisions because of the small sample size in West South Central (*n* = 11 participants).

**Table 1:** Participant Characteristics. Data from the Environmental influences on Child Health Outcome (ECHO) Cohort^a^

	(*N* = 8035) Mean (SD) or *N* (%)^b^
*Maternal Characteristics*	
age at birth, years, mean (SD)	30.2 (5.7)
% missing	1.3
*Educational Level*, *n* (%)	
less than high school	302 (4.3)
high school degree, GED, or equivalent	808 (11.5)
some college, associate’s degree	1892 (27.0)
bachelor’s degree and above	4014 (57.2)
% missing	12.7
*Marital Status*, *n* (%)	
single, widowed, or separated	885 (18.1)
married or living with a partner	4003 (81.9)
% missing	39.2
pregnancy tobacco use, yes, *n* (%)	664 (9.5)
% missing	13.3
pregnancy alcohol consumption, yes, *n* (%)	1402 (21.3)
% missing	18.2
*Prepregnancy BMI*, *n* (%)	
healthy weight	3437 (49.5)
overweight	1776 (25.5)
obesity	1739 (25.0)
% missing	13.5
self-reported history of any psychiatric disorder, yes, *n* (%)	31.68 (55.7)
% missing	29.2
nulliparous, yes, *n* (%)	2298 (38.4)
% missing	25.6
*Urbanicity of Residential Address in Pregnancy*, *n* (%)	
nonmetropolitan	1079 (13.5)
metropolitan	6930 (86.5)
% missing	0.3
*Air Pollution Exposure, Averaged in Pregnancy*, *n* (%)	
PM_2.5_, μg/m^3^	9.27 (2.73)
NO_2_, ppb	21.76 (8.78)
O_3_, ppb	40.32 (5.54)
% missing	0
*Child Characteristics*	
ASD diagnosis, *n* (%)	444 (5.5)
% missing	0
SRS total raw score, median (25th, 75th)^c^	22 (11, 36)
% missing	0
age at SRS assessment, years, mean (SD)	9.4 (3.6)
% missing	0
*SRS Form*, *n* (%)	
preschool version	649 (8.1)
school-age version	7386 (91.9)
% missing	0
*Birth Year Category*, *n* (%)	
2000–2002	692 (8.6)
2003–2007	1068 (13.4)
2008–2012	3748 (46.6)
2013 or after	2527 (31.4)
% missing	0
*Birth Season*, *n* (%)	
spring	2045 (25.5)
summer	1986 (24.6)
autumn	2045 (25.5)
winter	1959 (24.4)
% missing	0
male sex, *n* (%)	4161 (51.8)
% missing	0
*Race and Ethnicity*, *n* (%)	
hispanic	1241 (15.5)
non-hispanic white	4599 (57.2)
non-hispanic black	1239 (15.4)
non-hispanic asian	245 (3.0)
non-hispanic other race	710 (8.8)
% missing	0

a ASD, autism spectrum disorder; BMI, body mass index; GED, General Educational Development; NO_2,_ nitrogen dioxide; O_3_, ozone; ppb, parts per billion; PM_2.5_, particulate matter an aerodynamic diameter of <2.5 μm; SD, standard deviation; SRS, Social Responsiveness Scale.

b Percentages are calculated based on valid observations and before imputations.

c Median, 25th and 75th percentiles.

Correlations between pregnancy-averaged air pollutants were small (*r* = −0.12 for O_3_ and PM_2.5_; *r* = −0.09 for O_3_ and NO_2_) to moderate (*r* = 0.44 for NO_2_ and PM_2.5_); therefore, we included all three pollutants in one model (see Figure S2 for the correlations between pollutants by trimesters in the overall sample and across census divisions). Primary analyses included pregnancy averages, and coefficients were reported per interquartile range (IQR) change in exposure levels—calculated based on the distribution in the overall study sample for each pollutant. To examine the potential impact of air pollution across the distribution of autism-related traits in the general population, we used quantile regression at different levels of SRS scores (10th, 50th, and 90th quantiles) following previous studies that used SRS.^33^ We used logistic regression to investigate the association of air pollution exposure with child ASD diagnosis. We additionally examined the associations with exposure averaged across each trimester of pregnancy and with child outcomes separately because of high correlations between trimester-specific measures. We also ran analyses with trimester exposures of all three pollutants simultaneously in one model to examine the associations with each trimester independent of exposures in other exposure windows.

We *a priori* selected confounders using the Directed Acyclic Graph and factors associated with air pollution and autism.^9–12,34,35^ As a proxy for unmeasured structural factors with influences on environmental exposures or outcomes, the models included urbanicity of residence (metropolitan/not metropolitan, based on census classifications by the National Center for Health Statistics), maternal self-reported educational level (<high school, high school diploma/General Educational Development, some college/associate’s degree, ≥ bachelor’s degree), marital status (married/living with partner vs other), pregnancy tobacco use and alcohol consumption (yes/no), medical record–reported age at birth, prepregnancy body mass index (harmonized across cohorts on the basis of values calculated with prepregnancy weight and maternal height, or self-reported prepregnancy BMI values and categorized as obesity, overweight, healthy weight), any psychiatric diagnosis (yes/no), parity vs nulliparity, child birth year (≤2002, 2003–2007, 2008–2012, ≥2013, to allow sufficient numbers of children in each category while accounting for major policy changes over time) and season, and child race and ethnicity (Hispanic, Non-Hispanic White, Non-Hispanic Black, other race). For models that included the SRS, child age at the time of SRS assessment (year) and the SRS version (preschool/school-age) were used to improve precision. We investigated effect modification by sex by testing for interaction (*p* < 0.05) and stratifying the analyses by child sex. We did not examine modification by race and ethnicity because of the small sample size for some groups in the census divisions.

We repeated modeling under the following conditions: (1) exclusion of participants from high-risk cohorts to determine whether findings were driven by children at high risk of autism, (2) restriction to the school-age SRS because of challenges in assessing ASD at younger ages, and (3) models with inverse probability weighting (IPW) of inclusion in the analysis. We also reran models in the sample stratified by birth year (before and after 2008) to determine whether time could act as an effect modifier.

All analyses were carried out with R version 4.3.3 ^36^ (R Project for Statistical Computing) between April and May 2024. The R *mice* package^37^ was used for multiple imputation, and meta-analyses were performed with the R *meta* package.^38^

## Results

Most of the child participants were non-hispanic white (57.2%). The majority of adult participants lived in metropolitan areas (86.5%) and were married/living with a partner (81.9%) (Table 1). Overall, the pregnancy averages for air pollution exposures were at or below national standards (mean [SD] PM_2.5_ = 9.3 [2.7] μg/m^3^; NO_2_ = 21.8 [8.8] ppb; O_3_ = 40.3 [5.5] ppb). The medians and IQRs for pollutants were 4.05 μg/m^3^ (7.23 to 11.28) for PM_2.5_, 11.46 ppb (15.21 to 26.67) for NO_2_, and 6.27 ppb (36.86 to 43.13) for O_3_. However, air pollutant concentrations varied substantially across the geographical areas (Figure S2). A total of 444 (5.5%) children had been diagnosed with ASD, with the highest diagnoses among participants in the Pacific census division (Table S1).

The results of the quantile regression models at the 10th, 50th, and 90th quantiles of the SRS showed no associations between prenatal PM_2.5_ exposure and autism-related traits at the median or 90th quantile (Figure 1, Supplementary Table S2). However, a higher exposure to PM_2.5_ was associated with higher SRS scores at the lower end of the SRS distribution (β per IQR increment in averaged pregnancy: PM_2.5_ = 0.74, 95% confidence interval [CI]: 0.09, 1.40, *I* ^2^=19%, *p* for heterogeneity = 0.28). We found no association between prenatal NO_2_ exposure and child autism-like traits in the meta-analysis; however, we observed high heterogeneity among divisions: higher NO_2_ exposure was associated with higher SRS at the median in residents of the mid-Atlantic, West North Central, and South Atlantic census divisions (Figure 2, Supplementary Table S2). Higher O_3_ was associated with higher SRS scores at the median (β per IQR increment = 0.83, 95% CI: 0.05, 1.61, *I* ^2^ = 20%, *p* for heterogeneity = 0.27) and highest quantile of the SRS distribution (β = 2.19, 95% CI: 0.06, 4.32, *I* ^2^ < 1%, *p* for heterogeneity = 0.87), but not at the low end (Figure 3, Supplementary Table S2).

**Figure 1:**
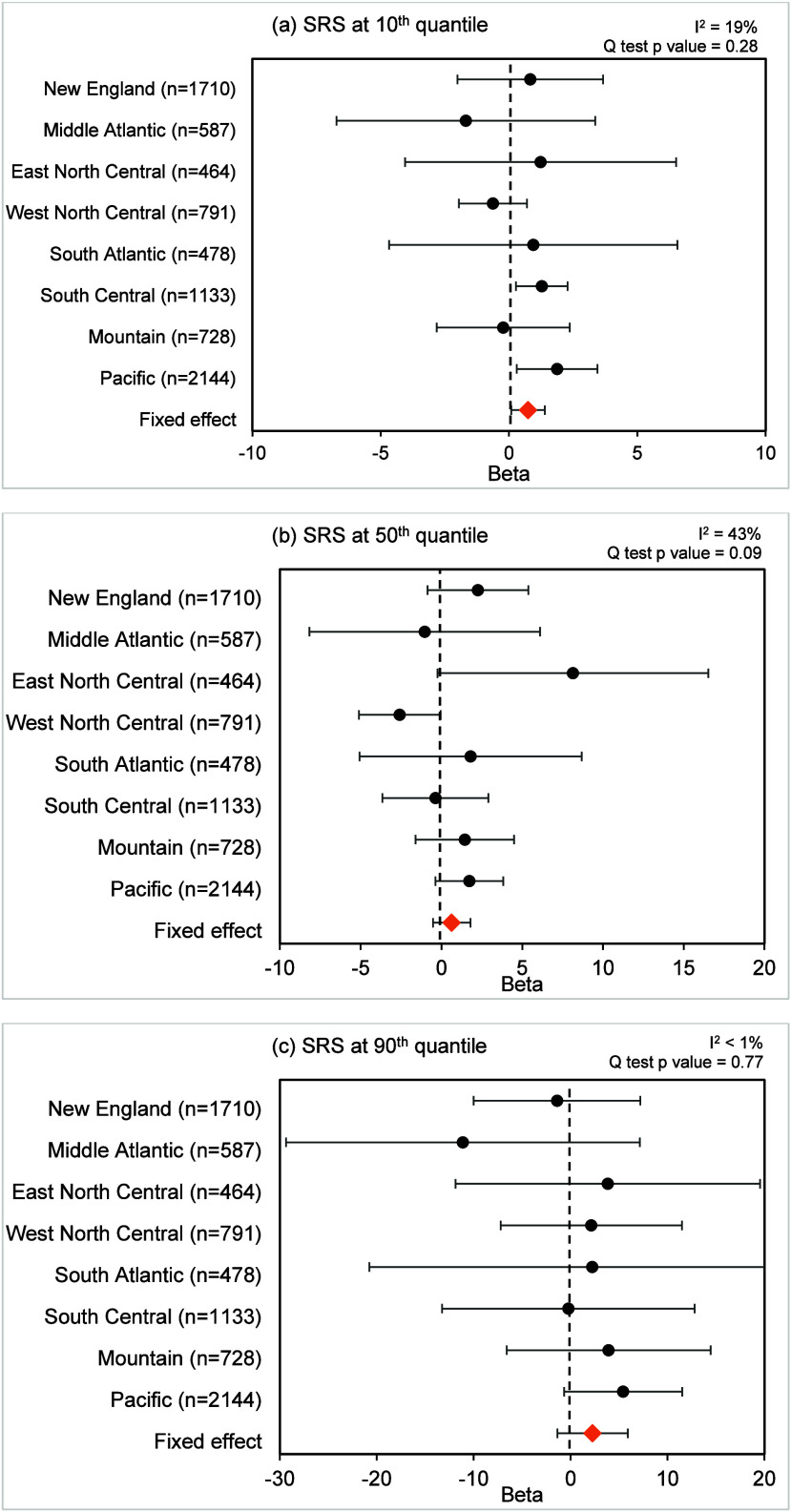
Associations between prenatal PM_2.5_ exposure and child SRS raw scores (*N* = 8,305). The *x* axis shows β coefficients and confidence intervals of associations in each census division and the meta-analysis (*y* axis). Coefficients are reported per interquartile range increase in exposure to PM_2.5_ (averaged across pregnancy) for SRS at (a) 10th quantile, (b) 50th quantile, and (c) 90th quantile. Higher SRS scores indicate higher autism-related traits. Models were adjusted for maternal age at birth, educational level, marital status, pregnancy tobacco use and alcohol consumption, prepregnancy body mass index, psychiatric disorder, parity, residential urbanicity, child birth year and season, child sex and age at assessment, child race and ethnicity, and SRS version. CI, confidence interval; PM_2.5,_ particulate matter an aerodynamic diameter of <2.5 μm; SRS, Social Responsiveness Scale.

**Figure 2:**
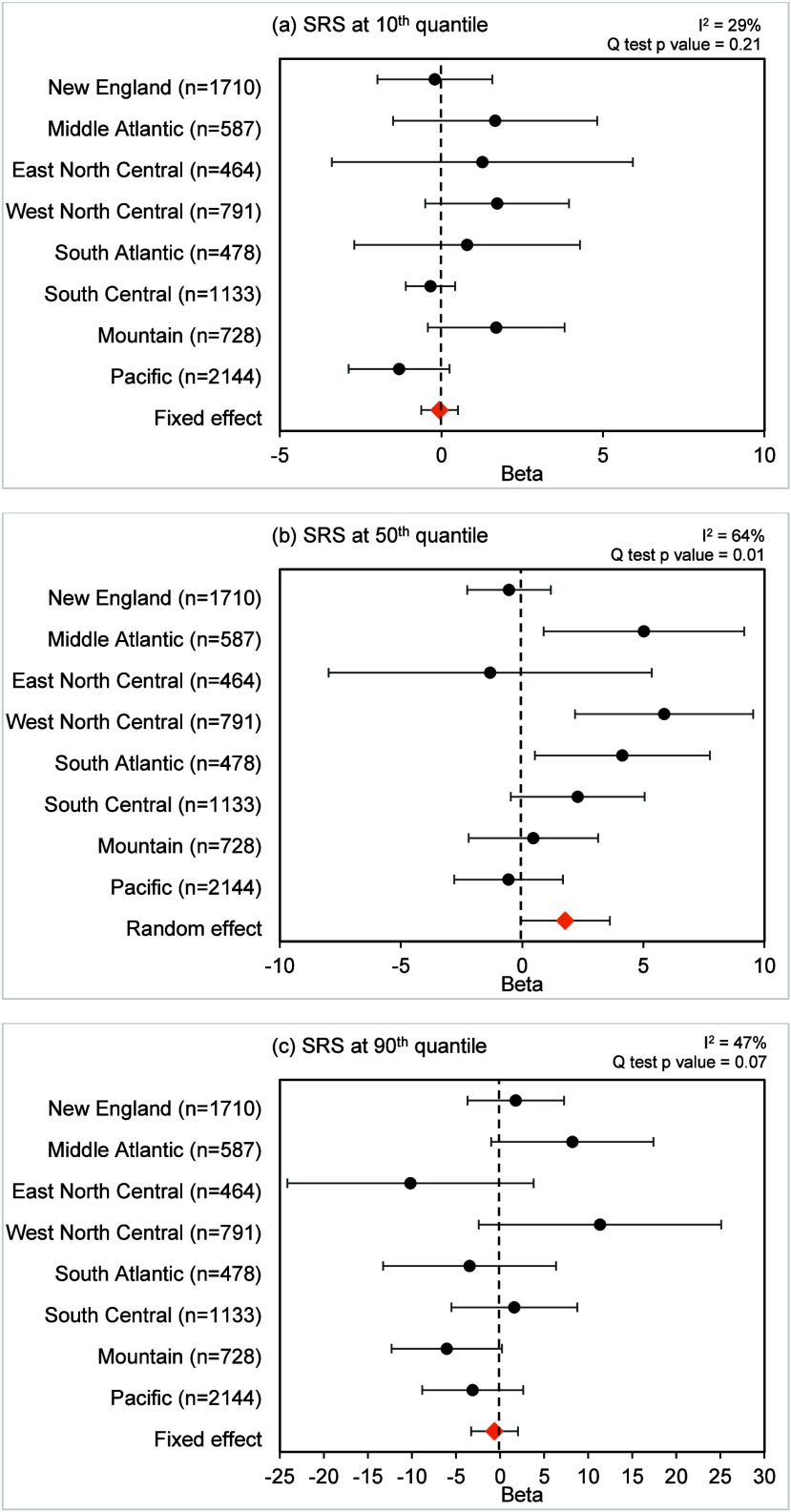
Associations between prenatal NO_2_ exposure and child SRS raw scores (*N* = 8,305). Data from the Environmental influences on Child Health Outcome (ECHO) Cohort. The *x* axis shows β coefficients and confidence intervals of associations in each census division and the meta-analysis (*y* axis). Coefficients are reported per interquartile range increase in exposure to NO_2_ (averaged across pregnancy) for SRS at (a) 10th quantile, (b) 50th quantile, and (c) 90th quantile. Higher SRS scores indicate higher autism-related traits. Models were adjusted for maternal age at birth, educational level, marital status, pregnancy tobacco use and alcohol consumption, prepregnancy body mass index, psychiatric disorder, parity, residential urbanicity, childbirth year and season, child sex and age at assessment, child race and ethnicity, and SRS version. CI, confidence interval; NO_2_, nitrogen dioxide; SRS, Social Responsiveness Scale.

**Figure 3:**
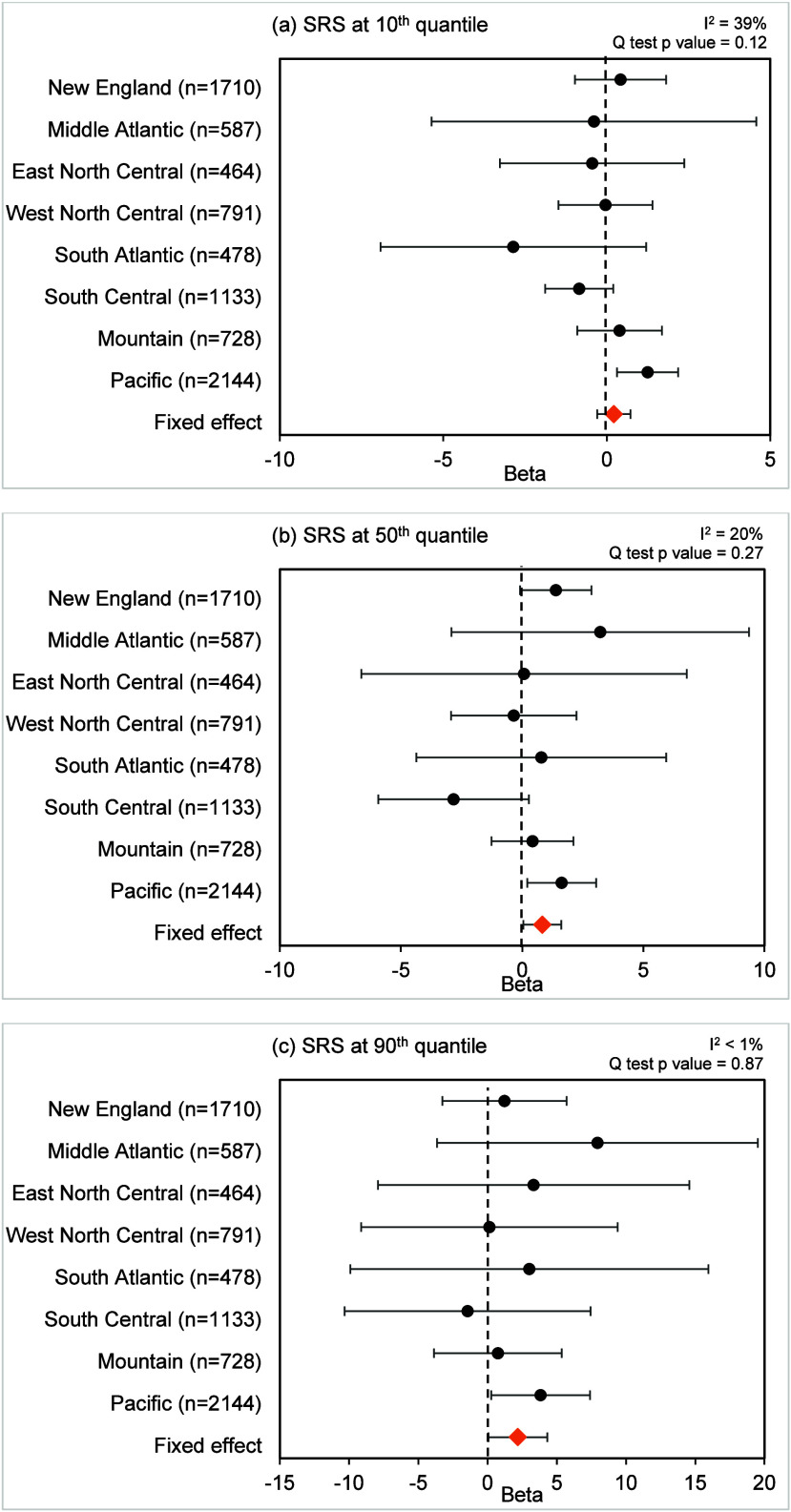
Associations between prenatal O_3_ exposure and child SRS raw scores (*N* = 8,305). Data from the Environmental influences on Child Health Outcome (ECHO) Cohort. The *x* axis shows β coefficients and confidence intervals of associations in each census division and the meta-analysis (*y* axis). Coefficients are reported per interquartile range increase in exposure to O_3_ (averaged across pregnancy) for SRS at (a) 10th quantile, (b) 50th quantile, and (c) 90th quantile. Higher SRS scores indicate higher autism-related traits. Models were adjusted for maternal age at birth, educational level, marital status, pregnancy tobacco use and alcohol consumption, prepregnancy body mass index, psychiatric disorder, parity, residential urbanicity, child birth year and season, child sex and age at assessment, child race and ethnicity, and SRS version. CI, confidence interval; O_3_, ozone; SRS, Social Responsiveness Scale.

Meta-analyses across census divisions showed no associations between PM_2.5_ and ASD diagnosis, with high heterogeneity among divisions (odds ratio [OR] per IQR increase = 0.70, 95% CI: 0.29, 1.69, *I* ^2^ = 76%, *p* for heterogeneity <0.001; Figure 4, Supplementary Table S2). Region-specific results showed that higher exposure to PM_2.5_ was associated with higher odds of ASD diagnosis in the Mountain (OR = 4.34, 95% CI: 1.29, 14.61) and Pacific (OR = 1.63, 95% CI: 1.23, 2.17) census divisions, but lower odds in the South Central (OR = 0.18, 95% CI: 0.05, 0.63) census division. We found no association between prenatal NO_2_ exposure and ASD diagnosis (OR = 1.14, 95% CI: 0.90, 1.45, *I* ^2^ = 14%, *p* for heterogeneity = 0.32). Higher O_3_ exposure during pregnancy was associated with higher odds of ASD (OR = 1.40, 95% CI: 1.19, 1.66, *I* ^2^ = 45%, *p* for heterogeneity = 0.08).

**Figure 4:**
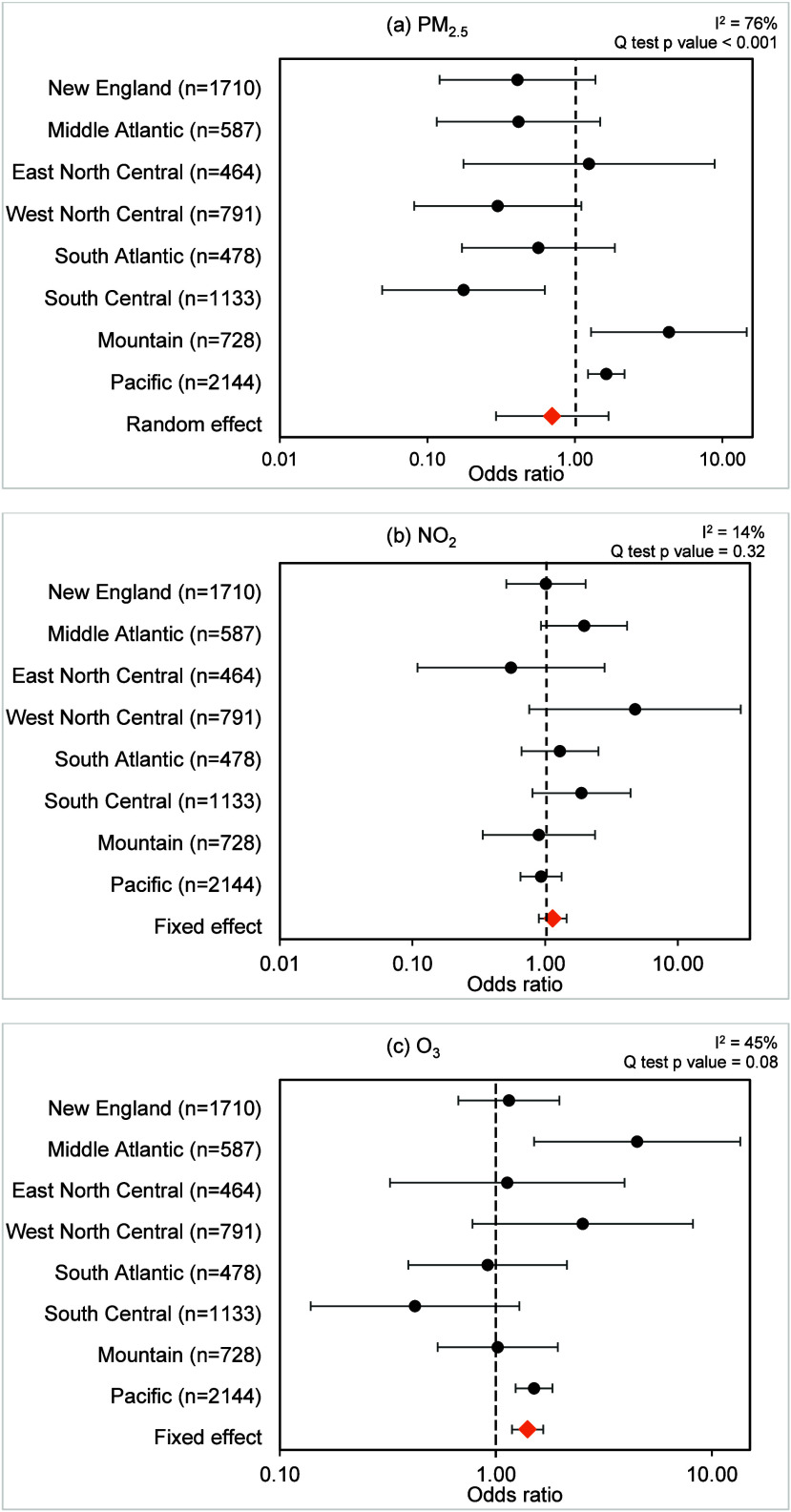
Associations between prenatal air pollution exposure and child ASD diagnosis (total *N* = 8,305; *n* for children with an ASD diagnosis = 444). Data from the Environmental influences on Child Health Outcome (ECHO) Cohort. The *x* axis shows odds ratios and confidence intervals of associations in each census division and the meta-analysis (*y* axis). Coefficients are reported per interquartile range increase in exposure (averaged across pregnancy) to (a) PM_2.5_, (b) NO_2_, and (c) O_3_. Models were adjusted for maternal age at birth, educational level, marital status, pregnancy tobacco use and alcohol consumption, prepregnancy body mass index, psychiatric disorder, parity, residential urbanicity, child birth year and season, child sex, and child race and ethnicity. ASD, autism spectrum disorder; CI, confidence interval; NO_2_, nitrogen dioxide; O_3,_ ozone; PM_2.5_, particulate matter an aerodynamic diameter of <2.5 μm.

In trimester-specific analyses, PM_2.5_ exposure in the first trimester was associated with higher SRS scores on average (Table S3). There were indications that the association between O_3_ exposure and outcome were driven by exposure in trimesters 1 and 3 (Table S3 for associations with SRS and Table S4 for associations with ASD diagnosis).

Although the interaction *p* values were not significant for any exposure, we observed some differences in the stratified analysis (Table S5). Females with higher prenatal exposure to PM_2.5_ had higher odds of ASD, but the association was not present in males. Males with higher NO_2_ exposure had higher SRS scores, but the association was not present in females (β for SRS at 50th quantile in males = 1.78, 95% CI: 0.28, 3.28; β for SRS at 50th quantile in females = 0.70, 95% CI: −1.93, 3.32). Males prenatally exposed to O_3_ also had higher odds of ASD (OR = 1.35, 95% CI: 1.11, 1.63), with a greater magnitude of association among females (OR = 1.58, 95% CI: 1.06, 2.36).

After excluding participants enrolled in high-risk cohorts, we found no associations between air pollutants and autism outcomes in the meta-analysis (β per IQR increment for SRS at the median = 0.47, 95% CI: −0.38, 1.33; OR for ASD = 0.91, 95% CI: 0.67, 1.23; Table S6). Region-specific data showed associations between PM_2.5_ exposure and ASD diagnosis: higher PM_2.5_ exposure was associated with higher odds of ASD diagnosis in the Mountain census division (OR = 4.56, 95% CI: 1.32, 15.71) but lower odds of ASD in Middle Atlantic (OR = 0.08, 95% CI: 0.01, 0.60), West North Central (OR = 0.17, 95% CI: 0.04, 0.77), and South Central (OR = 0.18, 95% CI: 0.05, 0.62) census divisions. PM_2.5_ exposure was associated with higher SRS in the Pacific census division (β per IQR increment for SRS at the median = 3.08, 95% CI: 1.09, 5.07) but lower SRS in the West North Center census division (β per IQR increment for SRS at the median = −4.52, 95% CI: −6.88, −2.17). Additionally, limiting the analyses to the SRS school-age version attenuated the associations (OR for O_3_ = 1.32, 95% CI: 0.78, 2.21; Table S7). Findings in the weighted analyses using IPW were consistent with the main analysis (Table S8). Results of analysis stratified by birth year showed no effect modification by time (data not shown).

## Discussion

Using a spatiotemporal model on a national scale to estimate prenatal exposure to three common air pollutants, this study provides evidence that low-level exposure to O_3_ may be a risk factor for autism. Prenatal O_3_ exposure, which was generally below the current standard in the study areas, was consistently associated with higher scores of autism-like traits and ASD diagnosis. Heterogeneity was high among census divisions for PM_2.5_ and NO_2_. Associations between PM_2.5_ and an ASD diagnosis were mainly present in the South Central, Mountain, and Pacific census divisions, whereas associations between NO_2_ exposure and autism-like traits were present in the mid-Atlantic, West North Central, and South Atlantic census divisions. Overall, there was little evidence for an interaction between exposure and child sex. These findings were independent of confounders, such as sociodemographic factors, family history, and urbanicity of residential addresses.

Our findings on O_3_ and autism outcomes are in contrast to the meta-analyses that previously reported limited evidence for the association between prenatal exposure to O_3_ and autism diagnosis.^10,11^ However, several studies have reported associations between *in utero* exposure to O_3_ and other neurodevelopmental outcomes, such as early developmental delay and intellectual disability.^39,40^ Experimental models have shown that O_3_ exposure interferes with recovery mechanisms of neuronal damage and neuronal survival through influences on brain-derived neurotrophic factor and other proteins.^41,42^ The importance of this finding is that average O_3_ levels were substantially lower than current standards across the study areas. Several previous studies relied on EPA data with lower spatiotemporal resolution in O_3_ that could lead to imprecision.^43,44^ Additionally, the few studies included in the meta-analyses had inconsistent results with high heterogeneity in the design.^10,11^ Our findings were attenuated after we excluded high-risk cohorts (effect estimate for SRS changed from 0.83 to 0.47). This difference could be due to several factors, such as preterm infants experiencing shorter periods of exposure or those from high-risk families having higher SRS scores. Ground-level O_3_ is a byproduct of chemical reactions in combination with other meteorological factors, such as heat and sunlight. With the increasing threat of O_3_, concern is growing regarding its health effects and the exposure-associated burden.^45^ Brain toxicity from O_3_ exposure occurs through neuroinflammation and oxidative stress,^46^ which are widely studied in the etiology of autism, particularly during the prenatal period.^47^ Our findings add to the literature, supporting the need for mitigation of factors contributing to inflammatory processes and oxidative stress in pregnancy which, in turn, may alter neurodevelopment over time.

Associations with PM_2.5_ at the lowest end of the distribution of SRS scores suggest that the risk might be present across the distribution of autism-like traits in the general population and at exposure levels substantially lower than those in previous studies.^9–12^ However, average exposure levels in some divisions (i.e., South Central, South Atlantic, East North Central, and Middle Atlantic) were still above the current standard for PM_2.5_. We observed high heterogeneity across divisions, with associations highly driven by participants in the South Central and Pacific divisions, the former having exposure levels above standards. The Pacific division had a high percentage of children diagnosed with ASD (11%), a fact that could contribute to heterogeneity. Heterogeneity across divisions highlights the challenges of studying PM exposure in large multisite studies across a vast geographical area and underlies the need for further examination of PM components during pregnancy in relation to autism in offspring. Evidence from non-U.S. studies that have examined child cognitive and psychomotor functioning suggests that components relevant to motorized traffic pollution (e.g., iron) are particularly neurotoxic.^48,49^ A study conducted in Southern California (in the Pacific division, which has high traffic-related air pollution) reported associations between PM_2.5_ components, including elemental and black carbon, and ASD risk, but emphasized the heterogeneity across studies because of different PM composition exposure models.^50^ Future studies on PM components could inform regulatory strategies that target lowering PM levels beyond current standards. Findings on the association between PM_2.5_ exposure in the first trimester and higher SRS scores merit further research to understand why children’s brains may be more susceptible to pollutants at different stages of pregnancy. One possible explanation could be the role of mild thyroid hormone insufficiency, which is associated with both early gestation PM_2.5_ exposure and child ASD or autism symptoms.^3,51^ The finding of a higher susceptibility to PM_2.5_ exposure in females was in contrast to one systematic review that suggested males are more susceptible to PM.^12^ But, our finding should be interpreted with caution because we found no significant *p* value for interaction between exposures and child sex, highlighting the need for more studies with large sample sizes that could better assess sex differences.

Our meta-analysis revealed no association between prenatal NO_2_ exposure and child SRS scores or ASD diagnosis. These findings align with previously conducted meta-analyses, which concluded that prenatal exposure to NO_2_ was not associated with autism in children.^10,11^ Region-specific analyses, however, showed that in certain census divisions, such as Middle Atlantic, West North Central, and South Atlantic, higher NO_2_ exposure was associated with higher autism symptoms. Interestingly, NO_2_ exposure levels in these study areas were well below the current standards.

In our study population, 444 children out of 8,035 (1 in 18) had an ASD diagnosis. This prevalence is higher than (double) the national average of 1 in 36 children with an ASD diagnosis according to a 2024 report from the Centers for Disease Control and Prevention. Although participants excluded from this analysis because of missing exposure information were less likely to have an ASD diagnosis than the children included, the ECHO Cohort encompasses study sites from the general population, as well as case-control studies of children with ASD and cohorts that include siblings of children with autism, which might explain the higher than expected number of children with ASD in this population. Our sensitivity analysis that excluded high-risk cohorts showed no association in the meta-analysis with both SRS and ASD as outcomes, suggesting that the observed association in the full ECHO Cohort could be driven by inclusion of case-control studies of children with ASD or other participants at high risk of ASD (e.g., preterm infants). Different patterns of region-specific results, in particular with PM_2.5_, support the need for further investigations with larger sample sizes in each region.

Our study has several strengths, including a large nationwide sample with data on both ASD diagnosis and autism-related traits across the distribution in the population, use of a national-level spatiotemporal air pollution model, and incorporation of three common air pollutants. Air pollution data included changes in address during pregnancy, which allowed for detailed, time-specific exposure estimates. However, these findings should be interpreted considering several limitations. First, we may have exposure misclassification because we relied on daily estimates of air pollution exposure at residential addresses only; we did not have information on work or other addresses or data on time spent outdoors. However, our use of proxy exposures based on models of ambient air pollution could address issues, such as confounding and reverse causation, which are often introduced with individual-level data.^52^ Second, outcomes were generally reported by parents, and we did not have information from electronic health records or clinical assessments. Third, this sample was not representative of the full ECHO Cohort, and the excluded participants differed from the included participants. This difference could potentially lead to bias, particularly with differential exposure levels among two groups. Fourth, studies included in the meta-analysis did not have similar designs; for example, case-control studies of autism frequency-matched the controls to the autism cases, yet our analytical approach did not account for the differences in study designs. Fifth, we did not have information on season of conception and used birth season as a proxy for it. Lastly, we did not have information on components of PM, which could allow us to explore heterogeneity by source.

## Conclusions

This study adds to the scientific literature by showing the potential risk for autism-related outcomes even at low levels of air pollution. The findings for prenatal O_3_ exposure are particularly concerning given the global increase in O_3_ exposure-related burden. In the context of modifiable factors, even small reductions in prenatal air pollution exposure could have a significant impact on child neurodevelopment, signaling the need for policy change and improved pollution regulations. Future studies are recommended to examine air pollution impacts on neurodevelopmental outcomes such as autism in other windows of vulnerability for brain, including preconception and early childhood.

## Supplementary Material





## Notes

Figures S1–S3 and Tables S1–S8 (PDF)
Complete list of collaborators for the ECHO Cohort Consortium (XLSX)
