# Supplementary material for: Prenatal Air Pollution Exposure and Autism Spectrum Disorder in the ECHO Consortium
Source: Environ Health Perspect. 2026 Apr 22;134(3):324–34. doi: 10.1021/EHP.6c00106 (PMC13347653; doi:10.1021/EHP.6c00106)
Supplement: Supplementary file 1 [file hp6c00106_si_001.pdf]

SUPPLEMENTAL MATERIAL

**Figure S1.** Study Flowchart	**2**
**Figure S2.** Correlations between Air Pollutants in Pregnancy Overall and by Census Divisions	**3**
**Figure S3.** Median and Interquartile Range of Air Pollutants in Pregnancy by Census Divisions	**6**
**Table S1.** Participant Characteristics by Census Division	**8**
**Table S2.** Associations Between Prenatal Air Pollution Exposure and Autism Outcomes by Census Divisions	**10**
**Table S3.** Associations Between Prenatal Air Pollution Exposure and Child SRS Raw Scores by Trimester of Exposure	**12**
**Table S4.** Associations Between Prenatal Air Pollution Exposure and Child ASD Diagnosis Scores by Trimester of Exposure	**14**
**Table S5.** Associations Between Prenatal Air Pollution Exposure and Autism Outcomes by Child Sex.	**15**
**Table S6.** Associations Between Prenatal Air Pollution Exposure and Autism Outcomes, Excluding Participants of High-Risk Cohorts	**16**
**Table S7.** Associations Between Prenatal Air Pollution Exposure and the SRS Scale Using the SRS School Version	**17**
**Table S8.** Associations Between Prenatal Air Pollution Exposure and Autism Outcomes using Inverse Probability Weighting to Account for Exclusion Because of Missing Data in Exposure.	**18**

Figure S1. Study Flowchart

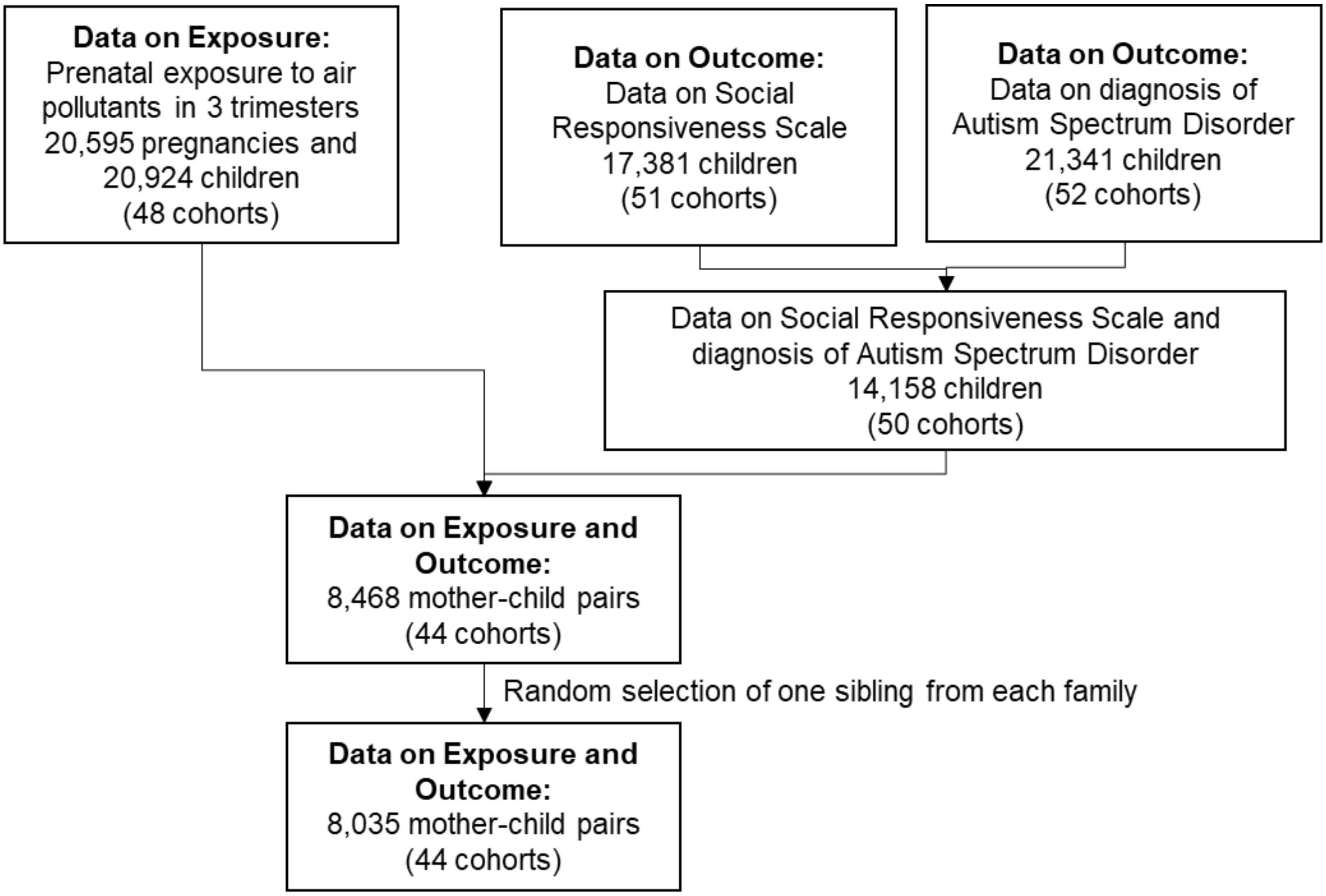

Figure S2. Correlations between Air Pollutants in Pregnancy Overall and by Census Divisions

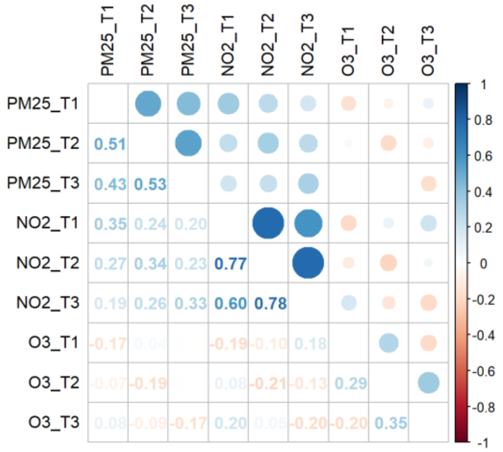

(a) Overall Cohort

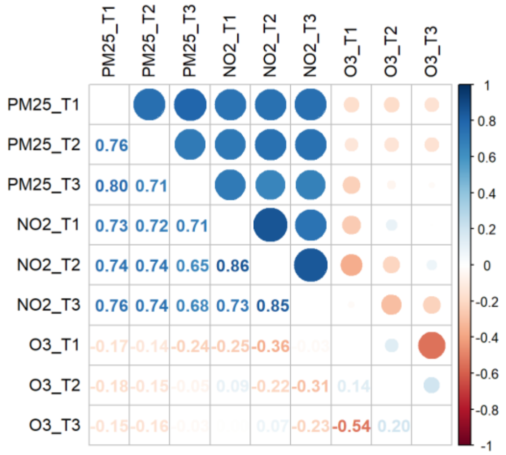

(b) New England

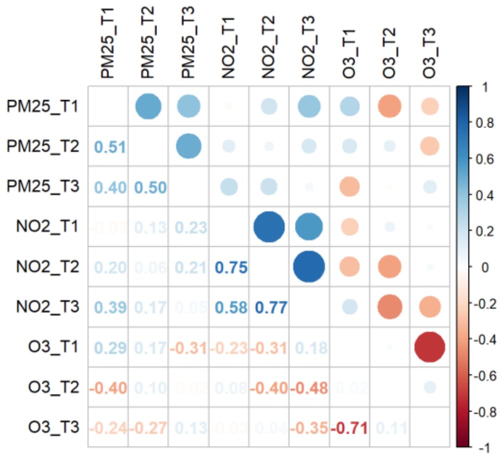

(c) Middle Atlantic

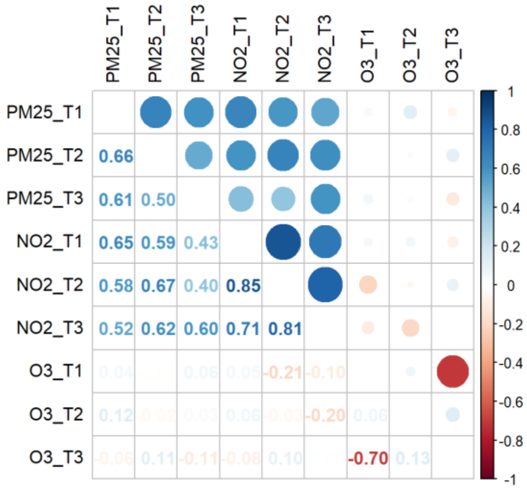

(d) East North Central

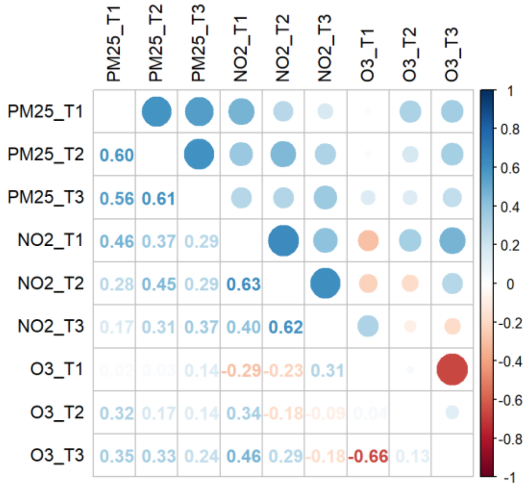

(e) West North Central

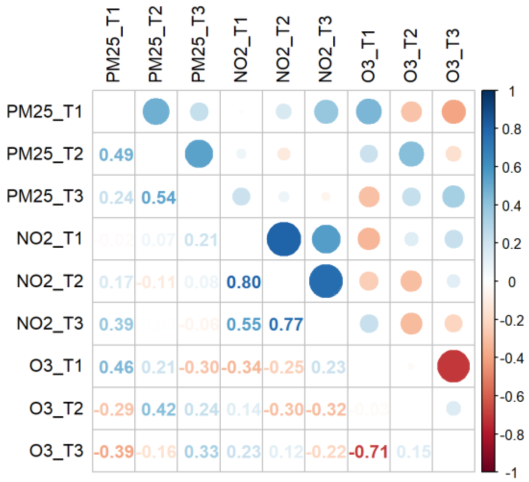

(f) South Atlantic

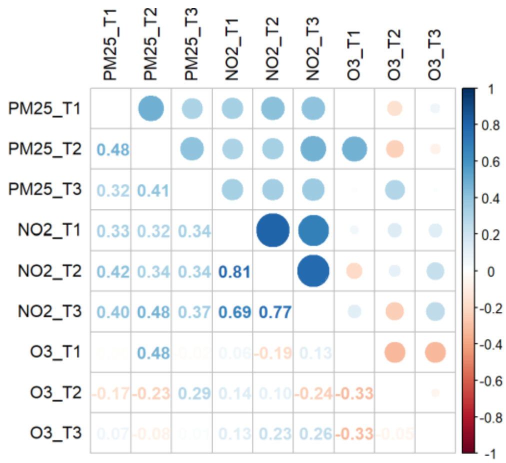

(g) South Centra

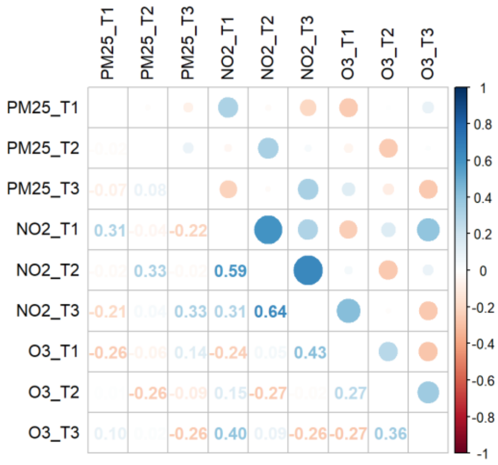

(h) Mountain

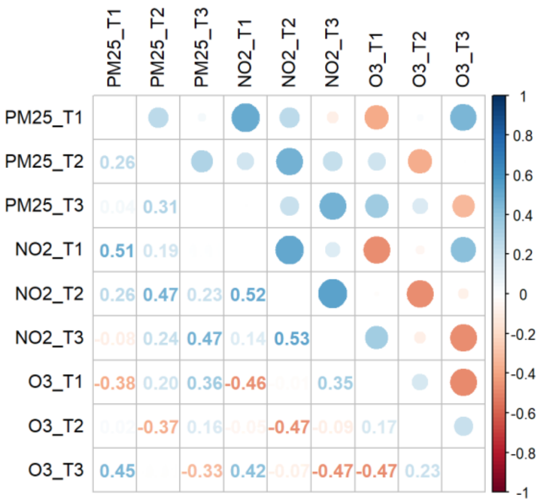

(i) Pacific

NO_2, nitrogen dioxide; O_3, ozone; $PM_{2.5}$, particulate matter an aerodynamic diameter of less than 2.5 μm; T_1: Trimester 1; T_2: Trimester 2; T_3: Trimester 3.

Figure S3. Median and Interquartile Range of Air Pollutants in Pregnancy by Census Divisions

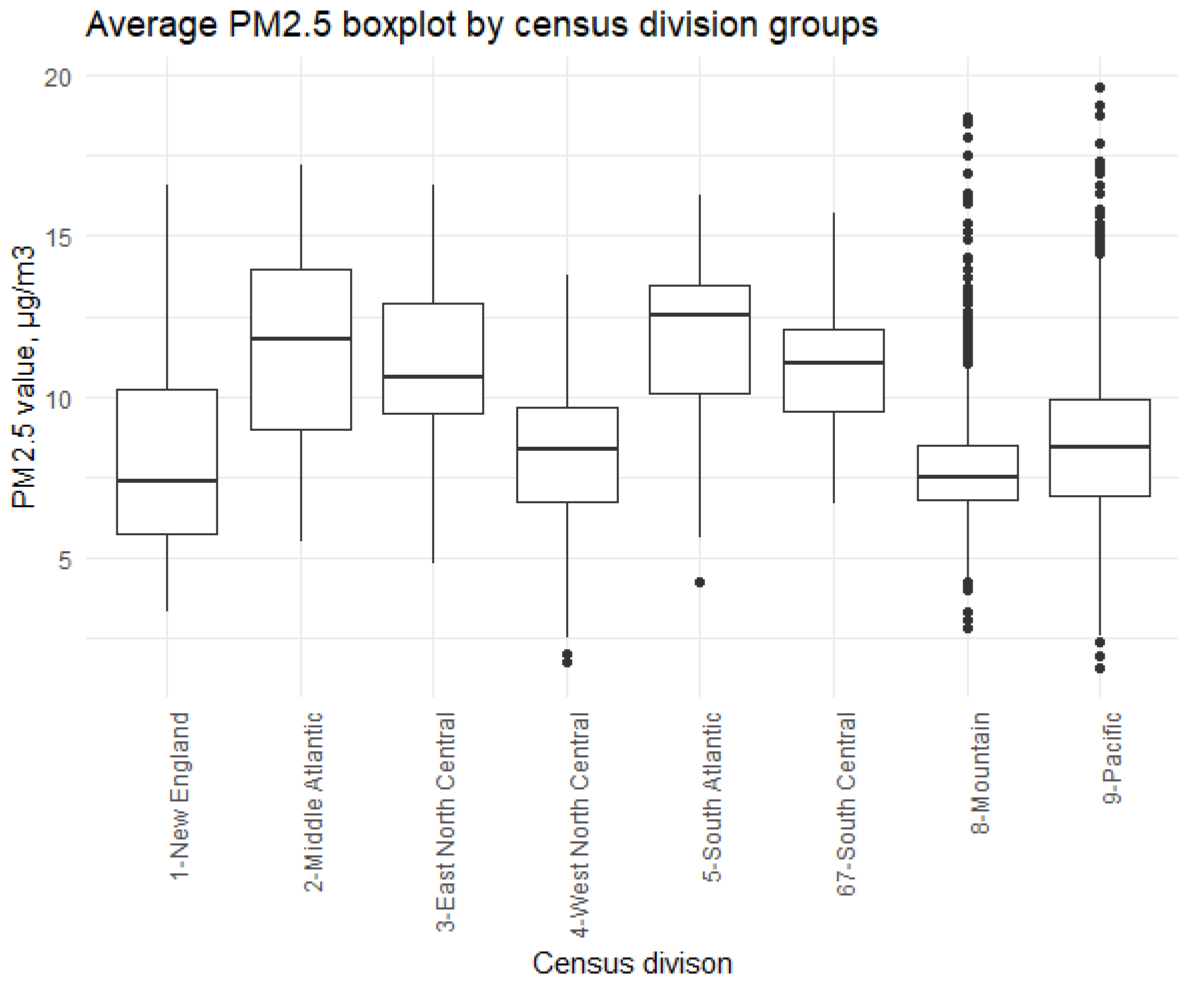

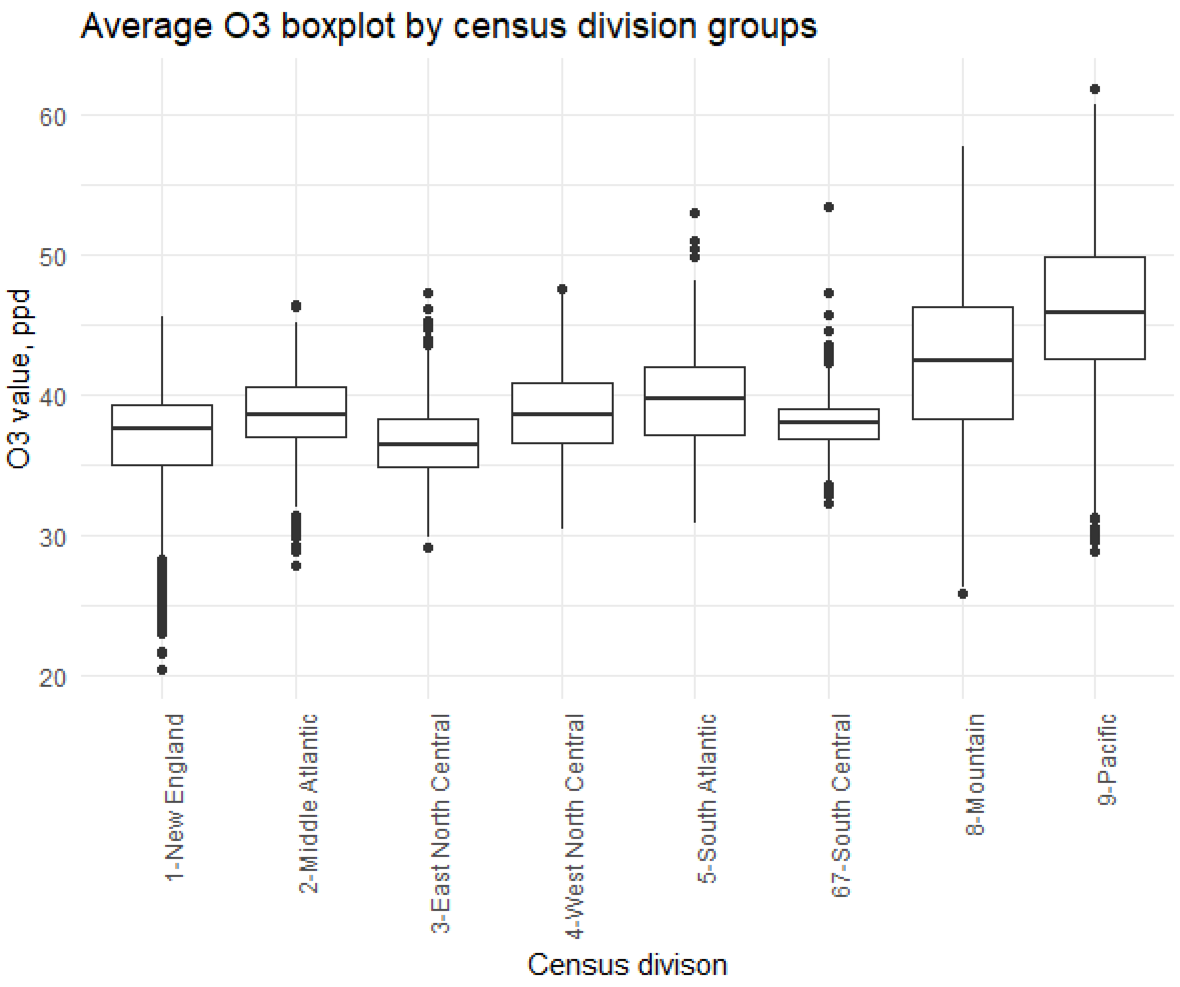

NO_2, nitrogen dioxide; O_3, ozone; $PM_{2.5}$, particulate matter an aerodynamic diameter of less than 2.5 μm

Table S1. Participant Characteristics by Census Division

	New England (N=1698)	Middle Atlantic (N=587)	East North Central (N=464)	West North Central (N=790)	South Atlantic (N=478)	South Central (N=1131)	Mountain (N=726)	Pacific (N=2135)
**Maternal characteristics**								
Age at enrollment, mean (SD), yrs	31.9 (5.1)	28.2 (6.2)	29.8 (5.3)	29.8 (5.0)	27.0 (6.6)	27.7 (5.5)	30.1 (5.1)	31.8 (5.4)
Educational level, n (%)								
Less than high school	34 (4.1)	47 (8.4)	25 (5.8)	25 (3.2)	41 (8.9)	67 (5.9)	11 (1.5)	50 (2.4)
High school degree, GED, or equivalent	58 (7.0)	130 (23.1)	66 (15.2)	63 (8.0)	98 (21.2)	175 (15.5)	36 (5.0)	179 (8.6)
Some college; associate degree	149 (18.1)	152 (27.0)	122 (28.2)	201 (25.5)	166 (35.9)	388 (34.3)	125 (17.4)	584 (28.1)
Bachelor's degree and above	583 (70.8)	233 (41.5)	220 (50.8)	498 (63.3)	158 (34.1)	500 (44.2)	547 (76.1)	1265 (60.9)
Marital status, n (%)								
Single, widowed or separated	177 (12.3)	77 (43.5)	56 (14.9)	44 (19.7)	59 (40.4)	278 (40.6)	61 (12.9)	126 (9.4)
Married or living with a partner	1267 (87.7)	100 (56.5)	321 (85.1)	179 (80.3)	87 (59.6)	407 (59.4)	411 (87.1)	1217 (90.6)
Pregnancy tobacco use, yes, n (%)	80 (4.9)	33 (15.1)	65 (16.7)	100 (14.0)	52 (21.4)	117 (10.4)	34 (4.8)	180 (9.4)
Pregnancy alcohol consumption, yes, n (%)	1540 (95.1)	185 (84.9)	325 (83.3)	616 (86.0)	191 (78.6)	1008 (89.6)	674 (95.2)	1743 (90.6)
Pre-pregnancy BMI, n (%)								
Healthy weight	887 (58.4)	151 (44.9)	155 (39.0)	334 (48.6)	110 (40.0)	469 (43.0)	393 (55.8)	923 (48.1)
Overweight	379 (24.9)	85 (25.3)	95 (23.9)	177 (25.8)	70 (25.5)	268 (24.6)	181 (25.7)	519 (27.1)
Obesity	254 (16.7)	100 (29.8)	147 (37.0)	176 (25.6)	95 (34.5)	352 (32.5)	<135	476 (24.8)
Maternal history of any psychiatric disorders, yes, n (%)	455 (43.0)	305 (63.1)	198 (59.6)	139 (54.5)	248 (60.5)	609 (62.5)	389 (60.1)	819 (54.2)
Nulliparous, yes, n (%)	449 (44.3)	77 (34.2)	97 (25.1)	249 (34.6)	82 (33.2)	296 (40.5)	252 (35.6)	789 (40.9)
Urbanicity of residential address in pregnancy, n (%)								
Non-metropolitan	727 (42.8)	26 (4.4)	26 (5.6)	67 (8.5)	163 (34.1)	21 (1.9)	11 (1.5)	38 (1.8)
Metropolitan	971 (57.2)	561(95.6)	438 (94.4)	723 (91.5)	315 (65.9)	1110 (98.1)	715 (98.5)	2097 (98.2)
**Pregnancy air pollution exposure, averaged across pregnancy**								
$PM_{2.5}$, μg/m^3	8.00 (2.71)	11.52 (2.68)	11.07 (2.34)	8.29 (2.23)	11.78 (2.15)	10.92 (1.56)	7.95 (2.05)	8.64 (2.40)
NO_2, pbb	21.52 (10.67)	23.10 (7.84)	23.60 (7.20)	15.48 (4.55)	20.64 (7.24)	19.51 (5.49)	33.96 (8.02)	20.69 (6.42)
O_3, pbb	36.65 (3.86)	38.53 (2.75)	36.63 (2.76)	38.74 (3.14)	39.65 (3.23)	37.92 (1.89)	42.21 (5.67)	45.95 (5.41)
**Child characteristics**								
ASD diagnosis, n (%)	36 (2.1)	38 (6.5)	14 (3.0)	18 (2.3)	37 (7.7)	43 (3.8)	17 (2.3)	241 (11.3)
SRS total raw score, median (IQR)	23.0 [14.0, 34.0]	27.0 [16.0, 42.0]	23.0 [12.0, 40.0]	12.00 [4.00, 29.00]	26.00 [14.00, 43.00]	9.00 [4.00, 23.00]	24.00 [15.00, 36.00]	26.00 [15.00, 41.00]
Age at SRS assessment, mean (SD), yrs	7.5 (3.3)	11.8 (4.4)	10.2 (4.4)	8.9 (2.2)	13.3 (4.5)	10.1(2.0)	8.4 (2.6)	9.0 (3.5)
SRS form, n (%)								

Preschool version	123 (7.2)	58 (9.9)	45 (9.7)	125 (15.8)	10 (2.1)	16 (1.4)	63 (8.7)	209 (9.8)
School version	1575 (92.8)	529 (90.1)	419 (90.3)	665 (84.2)	468 (97.9)	1115 (98.6)	663 (91.3)	1926 (90.2)
Birth year category, n (%)								
2002 or before	482 (28.4)	28 (4.8)	16 (3.4)	0 (0.0)	62 (13.0)	<5	<10	86 (4.0)
2003-2007	40 (2.4)	264 (45.0)	138 (29.7)	20 (2.5)	256 (53.6)	<90	<40	217 (10.2)
2008-2012	795 (46.8)	181 (30.8)	110 (23.7)	549 (69.5)	54 (11.3)	906 (80.1)	461 (63.5)	683 (32.0)
2013 or after	381 (22.4)	114 (19.4)	200 (43.1)	221 (28.0)	106 (22.2)	136 (12.0)	219 (30.2)	1149 (53.8)
Birth season, n (%)								
Spring	463 (27.3)	139 (23.7)	117 (25.2)	203 (25.7)	115 (24.1)	307 (27.1)	168 (23.1)	528 (24.7)
Summer	440 (25.9)	151 (25.7)	108 (23.3)	192 (24.3)	112 (23.4)	255 (22.5)	183 (25.2)	539 (25.2)
Autumn	415 (24.4)	154 (26.2)	127 (27.4)	196 (24.8)	123 (25.7)	275 (24.3)	195 (26.9)	552 (25.9)
Winter	380 (22.4)	143 (24.4)	112 (24.1)	199 (25.2)	128 (26.8)	294 (26.0)	180 (24.8)	516 (24.2)
Sex, male, n (%)	841 (49.5)	310 (52.8)	220 (47.4)	387 (49.0)	240 (50.2)	575 (50.8)	381 (52.5)	1196 (56.0)
Race and ethnicity, n (%)								
Hispanic	185 (10.9)	91 (15.5)	53 (11.4)	27 (3.4)	33 (6.9)	53 (4.7)	109 (15.0)	682 (32.0)
Non-Hispanic White	1245 (73.3)	360 (61.3)	259 (55.8)	583 (73.8)	154 (32.2)	517 (45.7)	529 (72.9)	943 (44.2)
Non-Hispanic Black	120 (7.1)	88 (15.0)	111 (23.9)	76 (9.6)	252 (52.7)	488 (43.1)	34 (4.7)	64 (3.0)
Non-Hispanic Asian	28 (1.6)	6 (1.0)	8 (1.7)	6 (0.8)	5 (1.0)	9 (0.8)	6 (0.8)	176 (8.2)
Non-Hispanic Other Race	120 (7.1)	42 (7.2)	33 (7.1)	98 (12.4)	34 (7.1)	64 (5.7)	48 (6.6)	269 (12.6)

Data for East South Central and West South Central have been combined due to small n.

ASD, autism spectrum disorder; BMI, body mass index: IQR, interquartile range; NO_2, nitrogen dioxide; O_3, ozone; pbb, parts per billion; $PM_{2.5}$, particulate matter an aerodynamic diameter of less than 2.5 μm; SD, standard deviation; SRS, Social Responsiveness Scale.

Table S2. Associations Between Prenatal Air Pollution Exposure and Autism Outcomes by Census Division.

	New England N=1710	Middle Atlantic N=587	East North Central N=464	West North Central N=791	South Atlantic N=478	South Central N=1133	Mountain N=728	Pacific N=2144
**SRS raw score, 50th quantile**								
				**β (95% CI)**				
$PM_{2.5}$, per IQR								
Unadjusted	-1.50 (-3.29, 0.28)	-5.49 (-8.51, -2.47)	4.08 (-3.85, 12.00)	-4.01 (-8.75, 0.73)	9.32 (4.14, 14.50)	-4.19 (-7.02, -1.36)	1.54 (-1.59, 4.67)	2.78 (0.47, 5.09)
Adjusted	2.26 (-0.86, 5.38)	-1.03 (-8.17, 6.11)	8.14 (-0.24, 16.52)	-2.58 (-5.11, -0.05)	1.81 (-5.07, 8.68)	-0.37 (-3.65, 2.91)	1.45 (-1.60, 4.50)	1.73 (-0.37, 3.83)
NO_2, per IQR								
Unadjusted	0.23 (-1.23, 1.70)	4.06 (0.62, 7.51)	-0.52 (-7.63, 6.59)	17.96 (12.57, 23.36)	5.86 (2.33,9.39)	3.22 (1.00, 5.45)	0.87 (-1.36, 3.11)	-2.97 (-4.98, -0.95)
Adjusted	-0.54 (-2.27, 1.19)	5.02 (0.89, 9.16)	-1.32 (-7.99, 5.35)	5.86 (2.18, 9.54)	4.14 (0.53, 7.75)	2.29 (-0.47, 5.05)	0.46 (-2.21, 3.14)	-0.56 (-2.80, 1.68)
O_3, per IQR								
Unadjusted	2.19 (1.02, 3.35)	3.66 (-0.48, 7.80)	-2.43 (-8.68, 3.83)	4.19 (0.08, 8.30)	-0.83 (-5.15, 3.49)	1.95 (-0.90, 4.80)	0.66 (-0.81, 2.14)	1.02 (0.07, 1.97)
Adjusted	1.39 (-0.08, 2.86)	3.22 (-2.92, 9.36)	0.08 (-6.64, 6.80)	-0.35 (-2.94, 2.24)	0.79 (-4.37, 5.94)	-2.83 (-5.94, 0.28)	0.43 (-1.27, 2.12)	1.63 (0.21, 3.06)
**ASD diagnosis**								
				**OR (95% CI)**				
$PM_{2.5}$, per IQR								
Unadjusted	1.65 (0.70, 3.93)	0.55 (0.32, 0.95)	1.00 (0.27, 3.70)	0.49 (0.18, 1.33)	0.29 (0.15, 0.53)	0.23 (0.09, 0.62)	2.16 (1.01, 4.63)	2.15 (1.68, 2.74)
Adjusted	0.41 (0.12, 1.38)	0.41 (0.12, 1.48)	1.25 (0.18, 8.85)	0.30 (0.08, 1.11)	0.57 (0.17, 1.86)	0.18 (0.05, 0.63)	4.34 (1.29, 14.61)	1.63 (1.23, 2.17)
NO_2, per IQR								
Unadjusted	1.19 (0.63, 2.23)	1.20 (0.71, 2.01)	0.69 (0.22, 2.23)	2.67 (0.75, 9.47)	1.62 (0.93, 2.81)	1.87 (0.89, 3.94)	0.70 (0.35, 1.40)	1.26 (0.93, 1.72)
Adjusted	1.01 0.51, 2.02)	1.97 (0.93, 4.15)	0.55 (0.11, 2.81)	4.76 (0.76, 29.86)	1.29 (0.66, 2.52)	1.88 (0.80, 4.40)	0.90 (0.34, 2.38)	0.93 (0.65, 1.33)
O_3, per IQR								
Unadjusted	1.10	2.48	0.97	1.03	1.24	0.53 (	0.92	1.76

	(0.63, 1.90)	(1.08, 5.69)	(0.29, 3.26)	(0.36, 2.95)	(0.62, 2.48)	0.18, 1.55)	(0.55, 1.56)	(1.47, 2.11)
Adjusted	1.15	4.51	1.13	2.52	0.92	0.42	1.02	1.50
	(0.67, 1.97)	(1.50, 13.54)	(0.32, 3.94)	(0.78, 8.17)	(0.39, 2.13)	(0.14, 1.28)	(0.54, 1.94)	(1.23, 1.83)

Coefficients are reported per IQR increase in exposure (averaged across pregnancy). Higher SRS scores indicated higher autism-related traits. Models were adjusted for maternal age at birth, educational levels, marital status, pregnancy tobacco use and alcohol consumption, pre-pregnancy body mass index, psychiatric disorder, parity, residential urbanicity, child birth year and season, child sex and age at assessment (for models with SRS), child race and ethnicity, and SRS version (for models with SRS).

*p<0.05

ASD, autism spectrum disorder; CI, confidence interval; IQR, interquartile range; $NO_{2,}$ nitrogen dioxide; OR, odds ratio; $O_{3,}$ ozone; $PM_{2.5,}$ particulate matter an aerodynamic diameter of less than 2.5 μm; SD, standard deviation; SRS, Social Responsiveness Scale.

Table S3. Associations Between Prenatal Air Pollution Exposure and Child SRS Raw Scores by Trimester of Exposure

	**Exposure in Trimester 1 β (95% CI) Q test *P* value for heterogeneity**	**Exposure in Trimester 2 β (95% CI) Q test *P* value for heterogeneity**	**Exposure in Trimester 3 β (95% CI) Q test *P* value for heterogeneity**
**SRS, 10th quantile**			
$PM_{2.5}$, per IQR			
Unadjusted	0.18 (-1.59, 1.94)<br><0.001	0.25 (-0.90, 1.41)<br>0.002	-0.09 (-0.54, 0.35)<br>0.74
Adjusted for covariates	0.40 (-0.55, 1.34)<br>0.01	0.14 (-0.35, 0.63)<br>0.09	-0.19 (-0.68, 0.31)<br>0.32
…Mutually adjusted for all three trimesters and covariates	0.43 (-0.11, 0.97)<br>0.29	-0.19 (-0.82, 0.44)<br>0.58	-0.22 (-0.80, 0.35)<br>0.77
NO_2, per IQR			
Unadjusted	0.73 (-0.55, 2.00)<br>0.01	0.85 (-0.92, 2.61)<br><.001	0.76 (-1.10, 2.63)<br><.001
Adjusted for covariates	0.04 (-0.51, 0.58)<br>0.60	0.64 (-0.65, 1.92)<br>0.01	0.30 (-0.85, 1.46)<br>0.01
Mutually adjusted for all three trimesters and covariates	-0.22 (-1.16, 0.73)<br>0.76	0.24 (-0.81, 1.29)<br>0.52	-0.34 (-1.22, 0.55)<br>0.17
O_3, per IQR			
Unadjusted	0.54 (0.09, 0.98)*<br>0.27	0.63 (0.25, 1.02)*<br>0.25	0.09 (-0.30, 0.48)<br>0.078
Adjusted for covariates	0.29 (-0.14, 0.73)<br>0.61	0.43 (-0.03, 0.88)<br>0.70	0.03 (-0.39, 0.44)<br>0.15
Mutually adjusted for all three trimesters and covariates	0.33 (-0.39, 1.05)<br>0.21	0.40 (-0.20, 1.01)<br>0.72	-0.14 (-0.69, 0.42)<br>0.44
**SRS, 50th quantile**			
$PM_{2.5}$, per IQR			
Unadjusted	-0.29 (-2.70, 2.13)<br><.001	0.08 (-3.04, 3.19)<br><.001	-0.46 (-2.39, 1.47)<br>0.001
Adjusted for covariates	1.10 (0.26, 1.94)*<br>0.42	0.32 (-0.59, 1.24)<br>0.06	0.28 (-1.44, 1.99)<br><0.001
Mutually adjusted for all three trimesters and covariates	0.86 (-0.09, 1.81)<br>0.77	-0.24 (-1.34, 0.85)<br>0.24	-0.03 (-0.96, 0.89)<br>0.64
NO_2, per IQR			
Unadjusted	3.74 (-0.58, 8.07)<br><.001	3.56 (-1.03, 8.14)<br><.001	2.74 (-1.97, 7.45)<br><.001
Adjusted for covariates	1.51 (-0.46, 3.48)<br>0.03	2.03 (-0.24, 4.31)<br>0.003	1.15 (-0.90, 3.19)<br>0.005
Mutually adjusted for all three trimesters and covariates	0.61 (-0.85, 2.06)<br>0.12	0.67 (-0.98, 2.33)<br>0.42	-0.14 (-1.59, 1.30)<br>0.29
O_3, per IQR			
Unadjusted	0.77 (0.09, 1.45)*<br>0.67	1.52 (-0.72, 3.76)<br><.001	0.72 (0.05, 1.40)*<br>0.469
Adjusted for covariates	0.51 (-0.15, 1.16)<br>0.70	0.54 (-0.13, 1.21)<br>0.15	0.36 (-0.31, 1.04)<br>0.45

Mutually adjusted for all three trimesters and covariates	1.07 (0.07, 2.08)* 0.59	0.06 (-0.86, 0.97) 0.95	0.60 (-0.35, 1.54) 0.80
**SRS, 90th quantile**			
$PM_{2.5}$, per IQR			
Unadjusted	0.78 (-3.93, 5.48) 0.02	1.21 (-1.52, 3.94) 0.23	-0.06 (-2.74, 2.63) 0.02
Adjusted for covariates	1.62 (-0.99, 4.23) 0.10	0.85 (-1.64, 3.34) 0.89	-1.04 (-5.45, 3.36) 0.04
Mutually adjusted for all three trimesters and covariates	1.60 (-1.27, 4.48) 0.44	0.05 (-2.76, 2.86) 0.71	-.57 (-3.29, 2.16) 0.67
NO_2, per IQR			
Unadjusted	2.26 (-5.74, 10.27) <.001	4.39 (-5.56, 14.35) <.001	2.16 (-6.68, 11.01) <.001
Adjusted for covariates	0.32 (-2.22, 2.87) 0.07	-1.34 (-3.99, 1.32) 0.19	-0.43 (-3.07, 2.20) 0.30
Mutually adjusted for all three trimesters and covariates	0.73 (-3.01, 4.47) 0.03	0.75 (-3.50, 4.99) 0.02	-2.79 (-6.58, 1.01) 0.29
O_3, per IQR			
Unadjusted	0.28 (-2.04, 2.59) 0.75	2.18 (-2.13, 6.48) 0.02	1.40 (-0.59, 3.39) 0.23
Adjusted for covariates	0.38 (-1.60, 2.35) 0.71	1.39 (-2.67, 5.44) 0.03	1.16 (-0.77, 3.10) 0.57
Mutually adjusted for all three trimesters and covariates	0.85 (-1.95, 3.65) 0.36	0.39 (-1.87, 2.66) 0.69	2.04 (-0.25, 4.32) 0.32

Coefficients are reported per IQR increase in exposure in each trimester separately. Higher SRS scores indicated higher autism-related traits. Models were adjusted for maternal age at birth, educational levels, marital status, pregnancy tobacco use and alcohol consumption, pre-pregnancy body mass index, psychiatric disorder, parity, residential urbanicity, child birth year and season, child sex and age at assessment, child race and ethnicity, and SRS version.

*p<0.05

CI, confidence interval; IQR, interquartile range; NO_2, nitrogen dioxide; O_3, ozone; $PM_{2.5}$, particulate matter an aerodynamic diameter of less than 2.5 μm; SRS, Social Responsiveness Scale.

Table S4. Associations Between Prenatal Air Pollution Exposure and Child ASD Diagnosis by Trimester of Exposure

	**Exposure in Trimester 1 OR (95% CI) Q test *P* value for heterogeneity**	**Exposure in Trimester 2 OR (95% CI) Q test *P* value for heterogeneity**	**Exposure in Trimester 3 OR (95% CI) Q test *P* value for heterogeneity**
**ASD diagnosis**			
$PM_{2.5}$, per IQR			
Unadjusted	0.79 (0.49, 1.26)<br><.001	0.82 (0.47, 1.46)<br><.001	0.83 (0.46, 1.50)<br><.001
Adjusted for covariates	1.17 (0.99, 1.39)<br>0.04	1.13 (0.95, 1.35)<br>0.07	0.87 (0.48, 1.59)<br><.001
Mutually adjusted for all three trimesters and covariates	1.08 (0.89, 1.30)<br>0.43	1.02 (0.83, 1.26)<br>0.81	1.14 (0.93, 1.39)<br>0.01
NO_2, per IQR			
Unadjusted	1.28 (1.06, 1.54)*<br>0.36	1.35 (1.11, 1.64)*<br>0.31	1.16 (0.96, 1.41)<br>0.49
Adjusted for covariates	1.11 (0.88, 1.40)<br>0.30	1.19 (0.95, 1.50)<br>0.63	0.95 (0.75, 1.19)<br>0.91
Mutually adjusted for all three trimesters and covariates	0.96 (0.68, 1.36)<br>0.35	1.29 (0.87, 1.93)<br>0.69	0.85 (0.60, 1.19)<br>0.97
O_3, per IQR			
Unadjusted	1.11 (0.78, 1.59)<br>0.01	1.29 (0.94, 1.76)<br>0.01	1.53 (1.32, 1.76)*<br>0.25
Adjusted for covariates	1.21 (1.03, 1.42)*<br>0.07	1.29 (1.11, 1.51)*<br>0.20	1.34 (1.14, 1.57)*<br>0.52
Mutually adjusted for all three trimesters and covariates	1.32 (1.04, 1.66)*<br>0.25	1.07 (0.87, 1.32)<br>0.44	1.54 (1.23, 1.93)*<br>0.17

Coefficients are reported per IQR increase in exposure in each trimester, from three separate models. Models were adjusted for maternal age at birth, educational levels, marital status, pregnancy tobacco use and alcohol consumption, pre-pregnancy body mass index, psychiatric disorder, parity, residential urbanicity, child birth year and season, child sex and race and ethnicity.

*$p<0.05$

ASD, autism spectrum disorder; CI, confidence interval; IQR, interquartile range; NO_2, nitrogen dioxide; OR, odds ratio; O_3, ozone; $PM_{2.5}$, particulate matter an aerodynamic diameter of less than 2.5 μm.

Table S5. Associations Between Prenatal Air Pollution Exposure and Autism Outcomes by Child Sex

ASD diagnosis	Males (n=4161) OR (95% CI) Q test *P* value for heterogeneity	Females (n=3874) OR (95% CI) Q test *P* value for heterogeneity	P for interaction with sex
$PM_{2.5}$, per IQR			
Unadjusted	0.80 (0.39, 1.64)<br><.001	0.71 (0.18, 2.72)<br><.001	0.24
Adjusted	0.60 (0.22, 1.64)<br>0.001	2.81 (1.51, 5.23)*<br>0.99	0.06
NO_2, per IQR			
Unadjusted	1.30 (1.03, 1.63)*<br>0.79	1.09 (0.70, 1.69)<br>0.67	0.45
Adjusted	1.15 (0.87, 1.52)<br>0.55	0.90 (0.48, 1.70)<br>0.73	0.20
O_3, per IQR			
Unadjusted	1.48 (1.25, 1.76)*<br>0.39	0.95 (0.33, 2.71)<br>0.02	0.95
Adjusted	1.35 (1.11, 1.63)*<br>0.51	1.58 (1.06, 2.36)*<br>0.09	0.40

SRS raw score, 50th quantile	β (95%CI) Q test *P* value for heterogeneity	β (95%CI) Q test *P* value for heterogeneity	
$PM_{2.5}$, per IQR			
Unadjusted	-0.91 (-5.52, 3.70)<br><.001	-0.09 (-3.62, 3.44)<br>0.005	0.32
Adjusted	0.39 (-1.43, 2.21)<br>0.96	1.19 (-0.59, 2.96)<br>0.24	0.19
NO_2, per IQR			
Unadjusted	4.75 (-1.01, 10.50)<br><.001	0.45 (-0.76, 1.65)<br><.001	0.07
Adjusted	1.78 (0.28, 3.28)*<br>0.36	0.70 (-1.93, 3.32)<br>0.04	0.15
O_3, per IQR			
Unadjusted	0.83 (-0.12, 1.78)<br>0.59	1.52 (0.52, 2.53)*<br>0.88	0.52
Adjusted	0.65 (-0.62, 1.91)<br>0.28	0.96 (-0.13, 2.05)<br>0.78	0.65

Coefficients are reported per IQR increase in exposure (averaged across pregnancy). Higher SRS scores indicated higher autism-related traits. Models were adjusted for maternal age at birth, educational levels, marital status, pregnancy tobacco use and alcohol consumption, pre-pregnancy body mass index, and any psychiatric disorder, parity, residential urbanicity as well as child's birth year and season, sex and age at assessment (for models with SRS), race and ethnicity, and version of the SRS (for models with SRS).

* $p < 0.05$

ASD, autism spectrum disorder, CI, confidence interval; IQR, interquartile range; NO_2, nitrogen dioxide; OR, odds ratio; O_3, ozone; $PM_{2.5}$, particulate matter an aerodynamic diameter of less than 2.5 μm; SRS, Social Responsiveness Scale.

Table S6. Associations Between Prenatal Air Pollution Exposure and Autism Outcomes, Excluding Participants of High-Risk Cohorts

ASD diagnosis	(n=6790) OR (95% CI) Q test *P* value for heterogeneity
$PM_{2.5}$, per IQR	
Unadjusted	0.69 (0.33, 1.41) 0.001
Adjusted	0.54 (0.18, 1.61) 0.003
NO_2, per IQR	
Unadjusted	1.18 (0.89, 1.56) 0.67
Adjusted	1.33 (0.93, 1.90) 0.68
O_3, per IQR	
Unadjusted	0.88 (0.68, 1.14) 0.69
Adjusted	0.91 (0.67, 1.23) 0.09
**SRS raw score, 50th quantile**	**β (95% CI) Q test *P* value for heterogeneity**
$PM_{2.5}$, per IQR	
Unadjusted	-1.59 (-5.38, 2.20) <.001
Adjusted	0.82 (-2.10, 3.74) <.001
NO_2, per IQR	
Unadjusted	2.33 (-2.63, 7.30) <.001
Adjusted	0.56 (-0.38, 1.51) 0.19
O_3, per IQR	
Unadjusted	1.46 (0.69, 2.24)* 0.35
Adjusted	0.47 (-0.38, 1.33) 0.43

Coefficients are reported per IQR increase in exposure (averaged across pregnancy). Higher SRS scores indicated higher autism-related traits. Models were adjusted for maternal age at birth, educational levels, marital status, pregnancy tobacco use and alcohol consumption, pre-pregnancy body mass index, psychiatric disorder, parity, residential urbanicity, child birth year and season, child sex and age at assessment (for models with SRS), child race and ethnicity, and SRS version (for models with SRS).

*$p<0.05$

ASD, autism spectrum disorder; CI, confidence interval; IQR, interquartile range; NO_2, nitrogen dioxide; OR, odds ratio; O_3, ozone; $PM_{2.5}$, particulate matter an aerodynamic diameter of less than 2.5 μm; SD, standard deviation; SRS, Social Responsiveness Scale.

Table S7. Associations Between Prenatal Air Pollution Exposure and the SRS Scale Using the SRS School Version

	(n=7386)
**SRS raw score, 50th quantile**	**β (95% CI)**<br>**Q test *P* value for heterogeneity**
$PM_{2.5}$, per IQR	
Unadjusted	0.16 (-4.11, 4.40)<br><.001
Adjusted	0.55 (-0.68, 1.70)<br>0.372
NO_2, per IQR	
Unadjusted	2.99 (-1.16, 7.10)<br><.001
Adjusted	1.56 (-0.74, 3.80)<br>0.01
O_3, per IQR	
Unadjusted	1.32 (0.64, 2.00)*<br>0.301
Adjusted	0.69 (-0.18, 1.50)<br>0.21

Coefficients are reported per IQR increase in exposure. Higher SRS scores indicated higher autism-related traits. Models were adjusted for maternal age at birth, educational levels, marital status, pregnancy tobacco use and alcohol consumption, pre-pregnancy body mass index, psychiatric disorder, parity, residential urbanicity, child birth year and season, child sex and age at assessment, and child race and ethnicity.
*p<0.05

ASD, autism spectrum disorder; CI, confidence interval; IQR, interquartile range; NO_2, nitrogen dioxide; OR, odds ratio; O_3, ozone; $PM_{2.5}$, particulate matter an aerodynamic diameter of less than 2.5 μm; SRS, Social Responsiveness Scale.

Table S8. Associations Between Prenatal Air Pollution Exposure and Autism Outcomes using Inverse Probability Weighting to Account for Exclusion Because of Missing Data in Exposure.

ASD diagnosis	(n=8035)<br>OR (95% CI)<br>Q test *P* value for heterogeneity
$PM_{2.5}$, per IQR	
Unadjusted	0.80 (0.38, 1.68)<br><0.001
Adjusted	0.69 (0.29, 1.66)<br><0.001
NO_2, per IQR	
Unadjusted	1.31 (1.06, 1.61)*<br>0.39
Adjusted	1.17 (0.91, 1.51)<br>0.25
O_3, per IQR	
Unadjusted	1.52 (1.30, 1.78)*<br>0.11
Adjusted	1.35 (1.13, 1.60)*<br>0.14

SRS raw score, 50th quantile	β (95% CI)<br>Q test *P* value for heterogeneity
$PM_{2.5}$, per IQR	
Unadjusted	0.31 (-3.84, 4.46)<br><.001
Adjusted	0.87 (-0.33, 2.07)<br>0.33
NO_2, per IQR	
Unadjusted	3.20 (-1.49, 7.89)<br><.001
Adjusted	1.87 (-0.43, 4.18)<br>0.01
O_3, per IQR	
Unadjusted	1.42 (0.81, 2.03)*<br>0.23
Adjusted	0.98 (0.16, 1.79)*<br>0.37

Coefficients are reported per IQR increase in exposure (averaged across pregnancy). Higher SRS scores indicated higher autism-related traits. Models were adjusted for maternal age at birth, educational levels, marital status, pregnancy tobacco use and alcohol consumption, pre-pregnancy body mass index, psychiatric disorder, parity, residential urbanicity, child birth year and season, child sex and age at assessment (for models with SRS), child race and ethnicity, and SRS version (for models with SRS).

*$p<0.05$

ASD, autism spectrum disorder; CI, confidence interval; IQR, interquartile range; NO_2, nitrogen dioxide; OR, odds ratio; O_3, ozone; $PM_{2.5}$, particulate matter an aerodynamic diameter of less than 2.5 μm; SD, standard deviation; SRS, Social Responsiveness Scale.
